# Nested Species Distribution Models of *Chlamydiales* in Ixodes ricinus (Tick) Hosts in Switzerland

**DOI:** 10.1128/AEM.01237-20

**Published:** 2020-12-17

**Authors:** Estelle Rochat, Séverine Vuilleumier, Sébastien Aeby, Gilbert Greub, Stéphane Joost

**Affiliations:** a Laboratory of Geographic Information Systems (LASIG), School of Architecture, Civil and Environmental Engineering (ENAC), Ecole Polytechnique Fédérale de Lausanne (EPFL), Lausanne, Switzerland; b La Source School of Nursing, University of Applied Sciences and Arts Western Switzerland (HES-SO), Lausanne, Switzerland; c Centre for Research on Intracellular Bacteria, Institute of Microbiology, University Hospital Centre and University of Lausanne, Lausanne, Switzerland; d Unit of Population Epidemiology, Division of Primary Care, Geneva University Hospitals, Geneva, Switzerland; e Group of Geographic Information Research and Analysis in Population Health (GIRAPH), Switzerland; University of Queensland

**Keywords:** *Ixodes ricinus*, species distribution modelling, ecological niche modelling, *Chlamydiales*, nested-niche, spatiotemporal variability

## Abstract

The tick Ixodes ricinus is the vector of various pathogens, including *Chlamydiales* bacteria, which potentially cause respiratory infections. In this study, we modeled the spatial distribution of *I. ricinus* and associated *Chlamydiales* over Switzerland from 2009 to 2019. We used a total of 2,293 ticks and 186 *Chlamydiales* occurrences provided by a Swiss Army field campaign, a collaborative smartphone application, and a prospective campaign. For each tick location, we retrieved from Swiss federal data sets the environmental factors reflecting the topography, climate, and land cover. We then used the Maxent modeling technique to estimate the suitability of particular areas for *I. ricinus* and to subsequently build the nested niche of *Chlamydiales* bacteria. Results indicate that *I. ricinus* habitat suitability is determined by higher temperature and normalized difference vegetation index (NDVI) values, lower temperature during the driest months, and a higher percentage of artificial and forest areas. The performance of the model was improved when extracting the environmental variables for a 100-m radius buffer around the sampling points and when considering the climatic conditions of the 2 years previous to the sampling date. *Chlamydiales* bacteria were favored by a lower percentage of artificial surfaces, drier conditions, high precipitation during the coldest months, and short distances to wetlands. From 2009 to 2018, we observed an extension of areas suitable to ticks and *Chlamydiales*, associated with a shift toward higher altitude. The importance of considering spatiotemporal variations in the environmental conditions for obtaining better prediction was also demonstrated.

**IMPORTANCE** Ixodes ricinus is the vector of pathogens including the agent of Lyme disease, the tick-borne encephalitis virus, and the less well-known *Chlamydiales* bacteria, which are responsible for certain respiratory infections. In this study, we identified the environmental factors influencing the presence of *I. ricinus* and *Chlamydiales* in Switzerland and generated maps of their distribution from 2009 to 2018. We found an important expansion of suitable areas for both the tick and the bacteria during the last decade. Results also provided the environmental factors that determine the presence of *Chlamydiales* within ticks. Distribution maps as generated here are expected to bring valuable information for decision makers in controlling tick-borne diseases in Switzerland and establishing prevention campaigns. The methodological framework presented could be used to predict the distribution and spread of other host-pathogen pairs to identify environmental factors driving their distribution and to develop control or prevention strategies accordingly.

## Abstract (precis)

Ixodes ricinus is the vector of pathogens including the agent of Lyme disease, the tick-borne encephalitis virus, and the less well-known *Chlamydiales* bacteria, which are responsible for certain respiratory infections. In this study, we identified the environmental factors influencing the presence of *I. ricinus* and *Chlamydiales* in Switzerland and generated maps of their distribution from 2009 to 2018. We found an important expansion of suitable areas for both the tick and the bacteria during the last decade. Results also provided the environmental factors that determine the presence of *Chlamydiales* within ticks. Distribution maps as generated here are expected to bring valuable information for decision makers in controlling tick-borne diseases in Switzerland and establishing prevention campaigns. The methodological framework presented could be used to predict the distribution and spread of other host-pathogen pairs to identify environmental factors driving their distribution and to develop control or prevention strategies accordingly.

## INTRODUCTION

Ixodes ricinus is the most common tick species in Switzerland and is known to be the vector of many pathogens, including the tick-borne encephalitis virus and the bacterium Borrelia burgdorferi, the agent of Lyme disease (1, 2). In 2015, Pilloux et al. showed that *I. ricinus* may also have the role of vector and even reservoir for *Chlamydiales* bacteria, especially *Rhabdochlamydiaceae* and *Parachlamydiaceae* (82). *Chlamydiales* is an order of strictly intracellular bacteria containing various bacterial pathogens or emerging pathogens associated with serious conditions for humans and animals, including respiratory tract infections and miscarriage (3–5). *Parachlamydiaceae* have been largely associated with free-living amoebae (6, 7) and are considered emerging agents of pneumonia in humans (8, 9). They have also been associated with miscarriage in ruminants (10, 11) and have been documented in roe deer and red deer, as well as in some rodents (12, 13). *Rhabdochlamydiaceae* species have mainly been described in association with arthropods, including Porcellio scaber, Blatta orientalis, and Ixodes ricinus (14–16). The pathogenic role of the *Rhabdochlamydiaceae* is still largely unknown, but they are suspected to cause newborn infections (17) and respiratory complications such as pneumonia (18).

Considering the potential threat to human health caused by pathogens associated with the tick Ixodes ricinus, studies have already investigated the influence of environmental factors on its presence or density. They have shown that the distribution and activity of *I. ricinus* are mainly influenced by temperature and humidity (19–22). Indeed, this tick species is prone to desiccation, and a relative humidity between 70% and 80% close to the soil is necessary for its survival (19, 20, 23). Its most favorable habitats may therefore be vegetation types able to maintain a high humidity level close to the soil, such as woodlands with thick vegetation litter (19, 22, 24).

In Switzerland, several studies analyzed the impact of environmental conditions on the activity or density of Ixodes ricinus. An early study done by Aeschlimann (19) indicated that *I. ricinus* distribution is mainly limited by the presence of a favorable vegetation cover, with a relative humidity close to or greater than 80% and an altitude lower than 1,500 m. Perret et al. (20) showed that the questing activity of ticks takes place from a temperature of 7°C and Hauser et al. (25) indicated that questing activity is largely reduced when the temperature exceeds 27°C. Jouda et al. (26) showed that in the region of Neuchâtel, the density of ticks decreases with altitude, which was confirmed by Gern et al. (27). However, this relationship was found to be the opposite in the Alps (Valais), which was explained by drier conditions at the lower altitude.

Bacterial communities within ticks are also known to be influenced by environmental conditions, notably through the modification of tick density, tick behavior, or the vector-host interactions (28–30). For example, B. burgdorferi is most likely found at lower altitudes (27), infects more ticks collected in forests than in pastures (29, 31), and may be favored by forest fragmentation (31, 32), whereas *Rickettsia* bacteria may be more prevalent in ticks in pasture sites showing a shrubby vegetation and a medium forest fragmentation (31). Environmental factors might provide us with critical information regarding bacterial distribution and thus potential threats to humans. However, nothing has yet been investigated regarding *Chlamydiales* bacteria.

Most studies described above analyzed the impact of environmental factors on the density or questing activity of ticks. None modeled across years the spatial distribution of Ixodes ricinus habitat suitability for the whole of Switzerland or the distribution of the *Chlamydiales* bacteria. In our study, we therefore aimed to build a model estimating the spatial distribution of the *I. ricinus* species from 2009 to 2019 in all of Switzerland using the Maxent modeling technique. Further, we also investigated the ecological factors that determine the distribution of *Chlamydiales* bacteria and the environmental factors that influence the presence of these bacteria within the tick host.

Modeling of *I. ricinus* distribution with Maxent has already been done at the scale of Europe (33), for an area including Europe, North Africa, and the Middle East (34), and in Romania (35). Environmental data used in these studies were extracted from Worldclim climatic data at a spatial resolution of 30 arc-seconds (approximately 1 km). These data summarized climatic conditions from 1950 to 2000. Therefore, in these studies as in many others (36–44), environmental data were extracted at a resolution that did not match the species ecology and more importantly the environmental conditions at the sampling dates. Our goals were thus to first build a model of higher spatial resolution (100 m) for Switzerland and then to use recent climatic data to characterize in detail the distribution of Ixodes ricinus and its associated *Chlamydiales* bacterial pathogen over Switzerland from 2009 to 2019. To better understand the importance of the environmental conditions surrounding the sampling points, and the conditions preceding the sampling date, we analyzed the performance of the model (i) across buffer zones around the sampling point and (ii) through different periods of time before the sampling date. Finally, we investigated the potential to use Maxent modeling to estimate the nested niche of a parasite within the ecological niche of its host.

## RESULTS

### Ixodes ricinus modeling.

**FIG 1:**
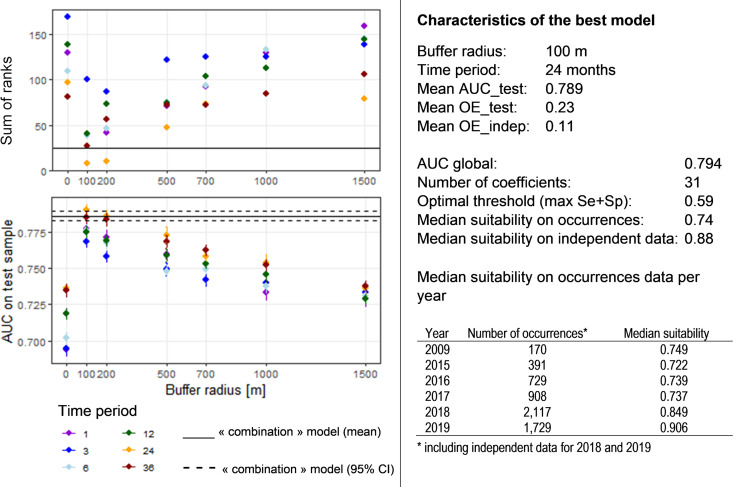
Performance of models predicting the habitat suitability for Ixodes ricinus. (Left) Values of the AUC_test_ and the sum of ranks as a function of the buffer radius and the time period (in months) considered for the extraction of the environmental variables. For the AUC_test_, the points indicate the mean value computed through the 20 runs and the lines correspond to the 95% confidence intervals. The “combination” model refers to the model derived by using for each environmental variable the combination of time period and buffer radius that best discriminates the tick’s presence from background locations (*t* test). (Right) Characteristics of the best model chosen according to the best values from the graphs at the left. OE_test is the omission error on the test samples and OE_indep the omission errors on the independent additional data available for 2018 and 2019.

**(i) Best model.** Among the 56 models tested with various parameters, the best one, according to the ranking procedure, was obtained with the following parameters: (i) background points selected below 1,500 m in altitude (corresponding to 6,049/10,000 points); (ii) a principal-component analysis (PCA) procedure to avoid correlated variables, with the components selected to retain 95% of the variance; (iii) a combination of linear and quadratic features; and (iv) a value of 5 for the regularization constant parameter. Details of the models tested and their corresponding evaluation parameters are available in Fig. S5 in the supplemental material. These parameters were then used to test the influence of the choice of buffer radius and time period on the performance of the models. Figure 1 shows the area under the receiver operating curve as computed on the testing data set (AUC_test_) and sum of ranks obtained for each combination. According to these results, the best model was obtained by extracting the environmental variables in a buffer with a 100-m radius around the sampling point and for the 2 years (24 months) preceding the sampling date. Note that the performance of the “combination” model was very similar, as were the performances of models obtained with an extraction for the 3 years preceding the sampling date and a buffer radius of 100 m and for the 2 years preceding the sampling date with a 200-m buffer radius. Moreover, we observed for each buffer radius that the models were more powerful when considering the variables extracted for the 2 or 3 years previous to the sampling date, instead of considering the conditions of the current year or even shorter time periods. Similarly, the models obtained by extracting the variables within buffers of 100-m or 200-m radius always outperformed the other models. Performance of models with variables extracted at the sampling coordinates only (radius = 0 m) was much lower than any buffer model, even those with a radius larger than 500 m. We retained the best model with variables extracted in a 100-m-radius buffer and for the 2 years preceding the sampling date (Fig. 1). The global area under the curve (AUC) obtained (with both the training and testing data) was 0.794 and the mean AUC_test_ obtained through the 20 runs was 0.789. The threshold maximizing the sum of sensitivity and specificity equaled 0.59. Using this threshold, the average omission error on the testing data set reached 23% and the omission rate on the independent data set was 11%. The model estimated 31 nonnegative coefficients. The median predicted suitability for all occurrences used in the model was 0.74 and the median suitability for independent occurrences from 2018 and 2019 was 0.88.

**FIG 2:**
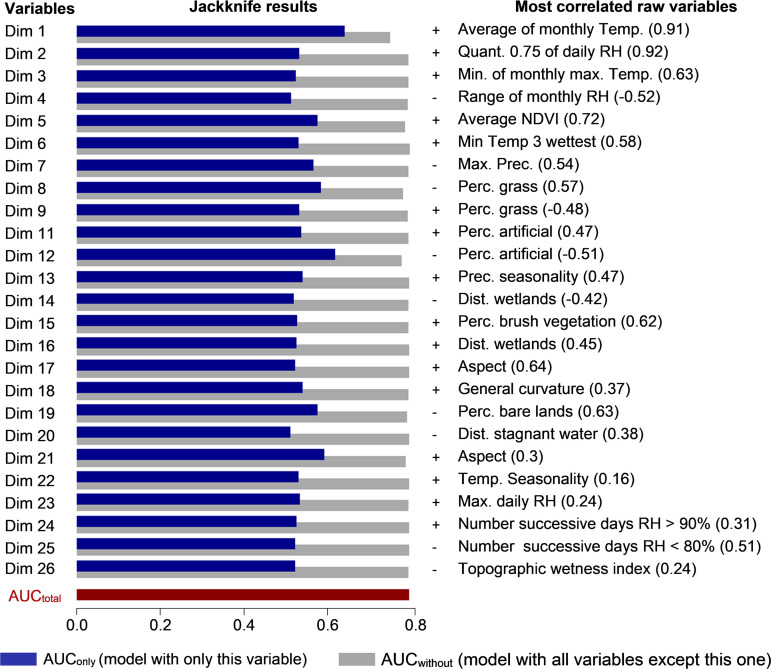
Jackknife results for the best model predicting the suitability of habitats for Ixodes ricinus. The variables Dim1 to Dim26 correspond to the components of the PCA needed to retain 95% of the variance. AUC_total_ corresponds to the performance of the model with all environmental variables, AUC_only_ the performance with only the environmental variable mentioned in the first column, and AUC_without_ being the performance with all the variables except the one mentioned. The column with +/− indicates the type of association between the component and the presence of Ixodes ricinus (with a positive association, the higher the value of the PCA dimension, the higher the suitability for ticks). The last column shows the raw environmental variable most correlated with the PCA dimension, with the value of the correlation indicated in parentheses (Temp, temperature; RH, relative humidity; Quant, quantile; Prec, precipitation; Perc, percentage).

**(ii) Effective variables.** The jackknife procedure (Fig. 2) indicated that the four variables containing the largest amount of important information not available in the other variables (lowest AUC_without_) were as follows: (i) dimension 1 (AUC_without_ = 0.748); (ii) dimension 12 (0.776); (iii) dimension 8 (0.780); and (iv) dimension 5 (0.784). The four variables containing the largest amount of important information by themselves (highest AUC_only_) were as follows: (i) the first dimension of the PCA (AUC_only_ = 0.641); (ii) dimension 12 (0.617); (iii) dimension 21 (0.591); and (iv) dimension 8 (0.582).

Dimension 1 of the PCA is strongly positively correlated with the average of the monthly mean temperatures (*r* = 0.91), which indicates that the presence of Ixodes ricinus is favored by higher mean temperature. Dimension 8 is moderately correlated with the percentage of herbs and grass vegetation (*r* = 0.57) and the mean temperature during the three driest consecutive months (*r* = 0.40). Its negative coefficient indicates that a higher percentage of herb and grass vegetation or higher temperature values during the driest months are less favorable for the presence of ticks. Dimension 12 is moderately negatively correlated with the percentage of artificial surfaces (*r* = −0.51) and positively correlated with the range of monthly normalized difference vegetation index (NDVI) (*r* = 0.35). This dimension is also negatively associated with habitat suitability for ticks, indicating that a higher percentage of artificial surfaces and a lower range of NDVI values are more favorable for the presence of *I. ricinus*. Finally, dimension 5 is positively correlated with the mean monthly NDVI (*r* = 0.72) and the minimum and maximum NDVI (*r* = 0.55 and 0.52, respectively) and is negatively correlated with the percentage of watery areas (r = −0.56). Its positive coefficient indicates that the areas with higher NDVI values and less water are more favorable for ticks.

**FIG 3:**
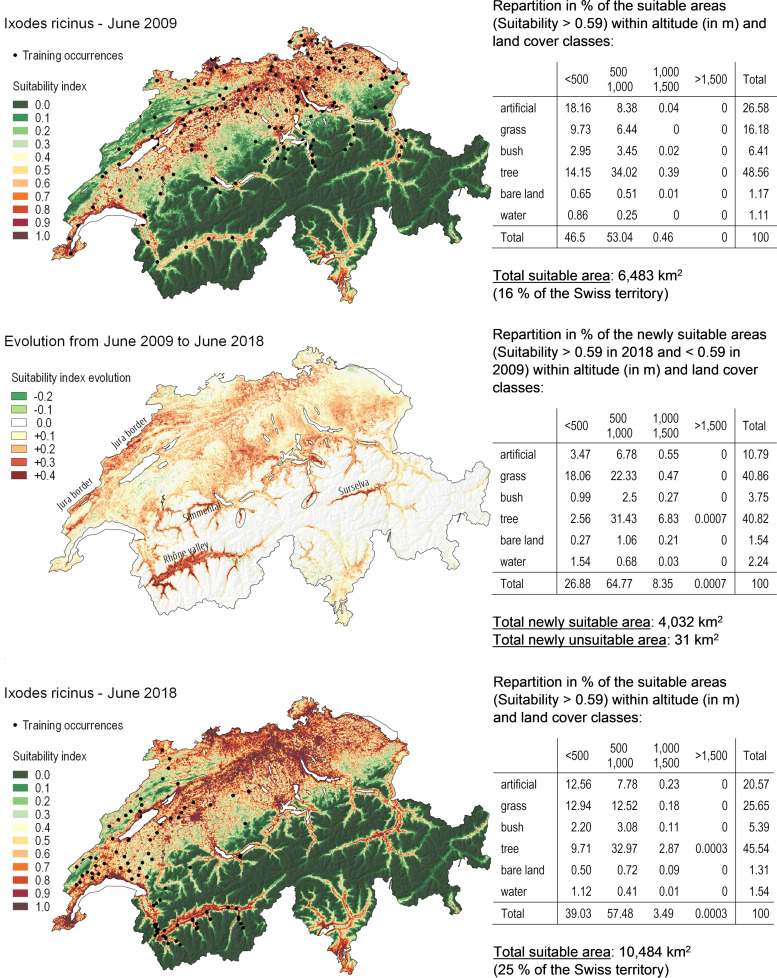
Suitability maps for Ixodes ricinus. Suitability map for Ixodes ricinus in June 2009 (upper panel) and June 2018 (lower panel) as predicted by the best model (i.e., with environmental variables extracted with a 100-m radius buffer and for the 2 years preceding the sampling date). The areas affected by transitions in suitability are represented in the middle panel.

**(iii) Distribution maps.** The maps of the distribution of Ixodes ricinus with suitability index values predicted by the model across Switzerland for June 2009 and June 2018 are shown in Fig. 3. The corresponding projections for the month of June in 2015, 2016, 2017, and 2019 are available in Fig. S6. Results for June 2009 show that 16% of the Swiss territory is predicted to be suitable for the presence of Ixodes ricinus when using the threshold maximizing the sum of specificity and sensitivity (threshold = 0.59). The suitable areas are mainly localized in land covered by tree vegetation (48.6% of all suitable areas); however, 26.6% are observed on hectares statistically classified as artificial surfaces. In addition, most of the suitable areas lay between 500 and 1,000 m in altitude (53.04%) or below 500 m (46.5%). Only 0.46% of the favorable area was found above 1,000 m in altitude.

In June 2018, 25% of the Swiss territory was predicted to be suitable for Ixodes ricinus (considering a threshold of 0.59). Between June 2009 and June 2018, the predicted suitable area increased by more than 4,000 km^2^ (Fig. 3) and only 31 km^2^ became unsuitable. The increased suitability was particularly pronounced in the Rhône Valley (Valais), in Surselva, in Simmental, on the Jura border, and in other lateral valleys of medium to high altitude (circles on the map). The evolution of the PCA components from 2009 to 2018 in these areas showed that the increase in suitability was generally associated with an increase in the values of dimension 1 (warmer temperature), an increase in dimension 5 (higher NDVI values), a decrease in dimension 12 (lower range of NDVI values), and a decrease in dimension 8 (temperature during driest months) in Valais and Jura (whereas this last dimension shows an increase of the values in Grisons). The new suitable areas comprised mainly grass and tree vegetation (40.8% each), with a large proportion (64.8%) located at an altitude between 500 and 1,000 m (corresponding, for example, to the altitude of the suitable hectares on the Jura border or in the Rhône valley). An increase in suitable areas mainly in forests was also observed between 1,000 and 1,500 m (8%). The model also predicted suitable areas above 1,500 m. These results therefore highlighted a spread of the favorable areas toward higher altitudes.

The distribution maps of Ixodes ricinus for the years 2015 to 2017 (Fig. S6) indicate a constant and drastic increase in suitability, which is highest between 2017 and 2018. Indeed, 15.7% of the Swiss territory was predicted as suitable in 2009, 16.8% in 2015, 16.2% in 2016, 17.6% in 2017, and 25.4% in 2018 (considering a threshold of 0.59 for suitable areas). Moreover, the map computed for 2019 predicted an important increase from 2018 to 2019, with 35% of the Swiss territory being predicted as suitable in 2019. The spread toward higher altitude was also observed between 2018 and 2019, with a maximal altitude for the favorable areas that reached 1,595 m in 2019. The results indicate that since 2018, there is a relatively high probability that ticks will reach such altitudes.

### *Chlamydiales* modeling.

**FIG 4:**
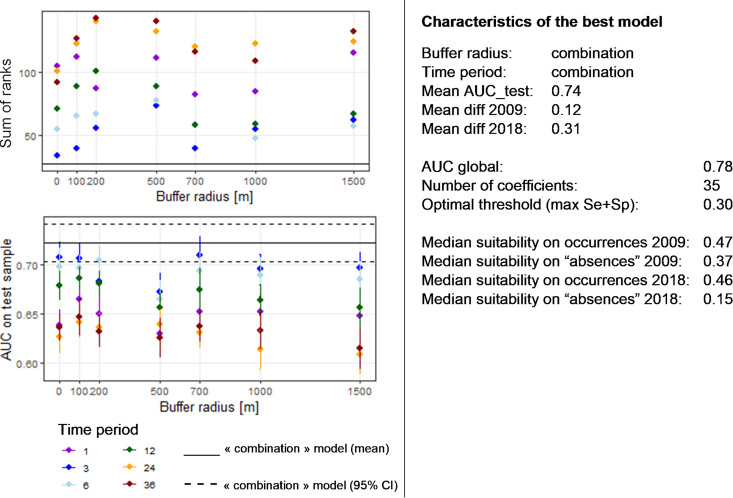
Performance of models predicting the suitability for *Chlamydiales*. (Left) Values of the AUC_test_ and the sum of ranks as a function of the buffer radius and the time period (in months) considered for the extraction of the environmental variables. For the AUC_test_, the points indicate the mean value computed over the 20 runs and the lines correspond to the 95% confidence intervals. The “combination” model refers to the model derived by using for each environmental variable the combination of time period and buffer radius that best discriminates the tick’s presence from background locations (*t* test). (Right) Characteristics of the best model chosen according to the graphs at the left. Mean diff 2009 and mean diff 2018 are the average differences between the mean suitability values predicted on *Chlamydiales* occurrence points and on “absences,” i.e., locations where no *Chlamydiales* were identified in 2009 and 2018, respectively.

**(i) Best model.** The best model for *Chlamydiales* bacteria, among the 60 models tested with various parameters, was obtained with the following parameters: (i) the “correlation-VIF” procedure to select uncorrelated variables; (ii) a combination of linear and quadratic features; and (iii) a value of 1 for the regularization constant parameter. The details of all models tested and their corresponding evaluation parameters are available in Fig. S7. As with the modeling of Ixodes ricinus, we then tested the influence of the choice of buffer radius and time period on the performance of the models. Figure 4 shows the AUC_test_ and sum of ranks obtained for each combination. According to these results, the “combination” model outperformed the other models. Unlike the results obtained for Ixodes ricinus, the models for *Chlamydiales* performed better when the variables were extracted for the 3 or 6 months preceding the sampling date than when considering 2 or 3 years before sampling (Fig. 4). In addition, the influence of buffer radius seemed to be much less pronounced than for the tick models. Accordingly, we retained the “combination” model. This model used 17 uncorrelated variables selected based on the “correlation/VIF” procedure. The list of these variables, as well as the results of the *t* test, can be found in Fig. S8. As the “combination” model aims to retain for each variable the best combination of buffer radius and time period, not all variables are selected using the same buffer radius or time period. Interestingly, we observed that the variables used in the model involved a buffer radius smaller than or equal to 200 m or else superior to 1 km (Fig. S8). The characteristics of the model are summarized in Fig. 4, on the right. The global AUC (with both training and testing occurrences) was 0.78 and the mean AUC_test_ obtained through the 20 runs was 0.74. The threshold maximizing the sum of sensitivity and specificity equaled 0.3. The mean suitability for *Chlamydiales* occurrence in 2009 was 0.47 and the mean suitability for sites where *Chlamydiales* were not identified in 2009 was 0.37. For 2018, the mean suitability on occurrence points was 0.46 and the suitability on sites where no *Chlamydiales* were identified was 0.15. The model estimated 35 nonnegative coefficients.

**FIG 5:**
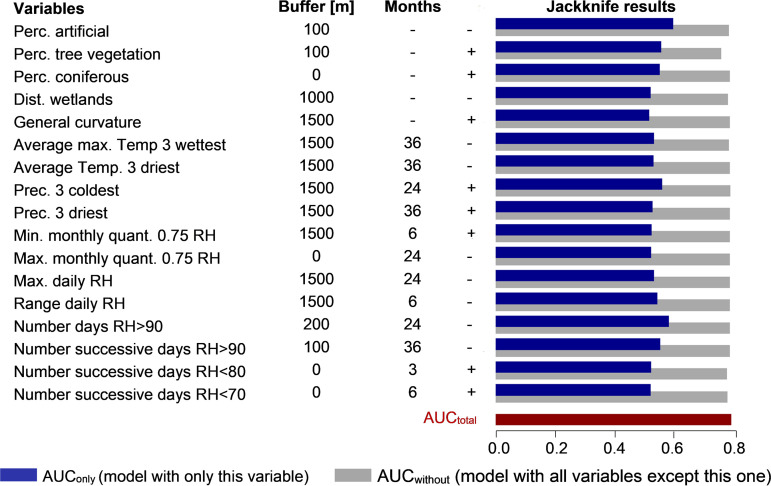
Jackknife results for the best model predicting the suitability for *Chlamydiales*. The column “Buffer” indicates the buffer radius around the sampling point and “Months” the number of months before the sampling date. The column with +/− indicates the type of association between the variable and the presence of *Chlamydiales* (with a positive association, the higher the value of the variable, the higher the suitability for *Chlamydiales*). AUC_total_ corresponds to the performance of the model with all environmental variables, AUC_only_ the performance with only the environmental variable mentioned in the first column, and AUC_without_ the performance with all the variables except the one mentioned. Perc, percentage; Temp, temperature; Prec, precipitation; quant. 0.75, quantile 0.75; RH, relative humidity.

**(ii) Effective variables.** The four variables containing the largest amount of important information that was not available in the other variables (lowest AUC_without_) were (Fig. 5) (i) the percentage of tree vegetation in a 100-m buffer (AUC_without_ = 0.75); (ii) the number of successive days with a relative humidity inferior to 80% during the 3 months preceding sampling (0.77); (iii) the number of successive days with a relative humidity inferior to 70% during the 6 months preceding sampling (0.77); and (iv) the distance to wetlands within a buffer of 1 km (0.77). The four variables containing the largest amount of important information by themselves (highest AUC_only_) were (i) the percentage of artificial surfaces in a 100-m buffer (AUC_only_ = 0.59); (ii) the number of days with a relative humidity superior to 90% in a 200-m buffer during the 2 years preceding the sampling date (0.57); (iii) the precipitation of the three coldest months in a 1.5-km buffer during the 2 years preceding the sampling (0.55); and (iv) the percentage of tree vegetation in a 100-m buffer around the sampling point (0.55).

The conditions favorable for *Chlamydiales* were thus characterized by the following: (i) a lower percentage of artificial surfaces around the sampling point (7.8% in average for the occurrences locations in a 100-m buffer versus 16.8% for the background locations); (ii) a higher percentage of tree vegetation (62.8% versus 53.1%); (iii) a lower number of days with a relative humidity superior to 90% during the 2 years preceding the sampling date (21.1 versus 25.2); (iv) a larger amount of precipitation during the coldest months (24.15 mm versus 20.7 mm); (v) a higher number of successive days with a relative humidity inferior to 80% during the three previous months (29.7 versus 27.1) and lower than 70% during the 6 previous months (16 versus 14.4); and finally, (vi) a shorter distance to wetlands (2.5 km versus 3.1 km).

**FIG 6:**
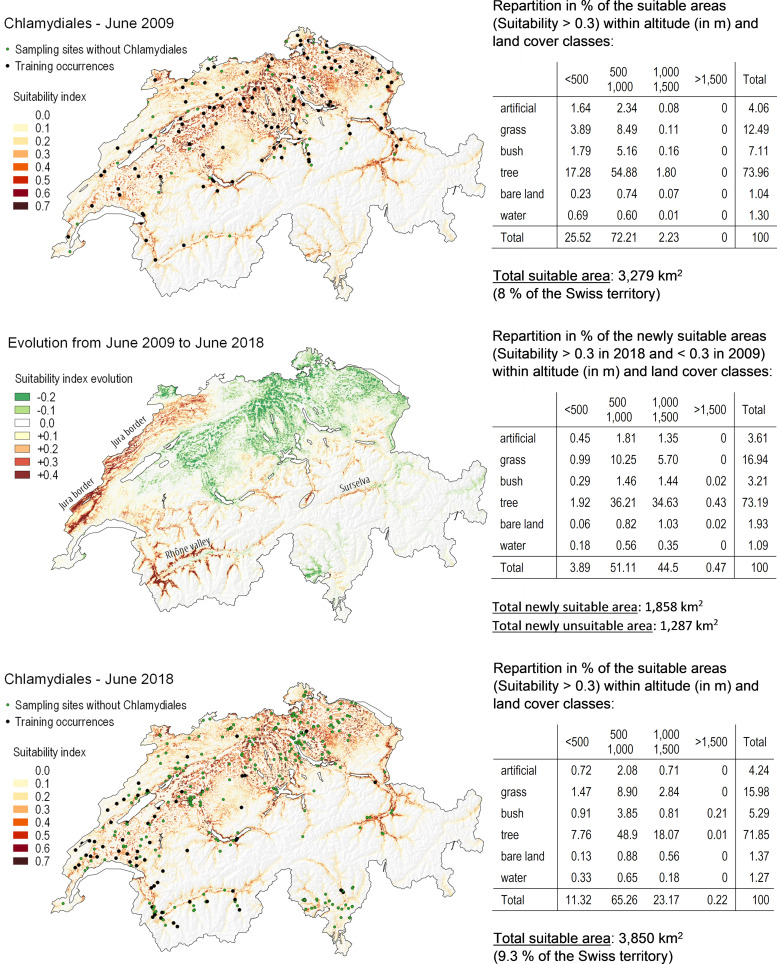
Suitability maps for *Chlamydiales* in June 2009 (upper panel) and June 2018 (lower panel) as predicted by the best model (i.e., with the “combination” set of environmental variables). The areas affected by transitions in suitability are represented in the middle panel. The green dots show sites where ticks were sampled but no *Chlamydiales* were identified. Note that, as explained in the text, these sites cannot be considered real “absences.”

**(iii) Distribution maps.** The distribution maps of *Chlamydiales* with values of suitability predicted by the model across Switzerland for June 2009 and June 2018 are shown in Fig. 6. In June 2009, 8% of the Swiss territory was predicted as favorable for *Chlamydiales* bacteria (using the threshold maximizing the sum of sensitivity and specificity). As the niche of the bacteria is nested within the niche of the tick, modeling suitability for *Chlamydiales* bacteria involved a multiplication by the suitability results for Ixodes ricinus. Therefore, the areas predicted to be unfavorable for the presence of the tick species are also predicted as weakly suitable for *Chlamydiales*. On the contrary, some areas predicted to be highly favorable for the presence of Ixodes ricinus in Fig. 3 did not match and showed very low values in Fig. 6. This is the case for the areas situated within urban settlements, for which a large portion was predicted to be suitable for ticks but not for *Chlamydiales*. Indeed, the distribution of the areas favorable for *Chlamydiales* within the various categories of land cover classes indicates that they are essentially observed in natural areas, covered either by tree (74%) or grass (12%) vegetation, and only 4% of them are observed in regions characterized by a large portion of artificial elements. When considering the altitudinal distribution, areas favorable for *Chlamydiales* seem to be essentially predicted in forests suitable for ticks, between 500 and 1,000 m in altitude. However, due to other factors influencing the model, notably the climatic conditions, 52% of those forests are also predicted to be unfavorable for the bacteria.

In June 2018, 9% of the Swiss territory was predicted as suitable for the presence of *Chlamydiales*. Between June 2009 and 2018, more than 1,850 km^2^ became newly suitable for *Chlamydiales*, as shown in Fig. 6. Some regions showed a sharp increase in suitability values (more than 0.4). However, more than 1,280 km^2^ was also becoming unsuitable. In 2018, the proportion of suitable area within land cover classes was close to what was observed in 2009; however, there was a clear spread toward higher altitude, with 23% of the favorable areas localized between 1,000 and 1,500 m, versus only 2% in 2009. Newly suitable areas match those of Ixodes ricinus in Fig. 3 (Rhône valley, Surselva, and the Jura border). The spread of favorable areas toward higher altitudes was also predicted, with 45% of the newly suitable hectares being localized between 1,000 and 1,500 m. Loss of suitable areas mainly occurred in the northwest part of Switzerland and appears to be associated with a decrease in precipitation during the three coldest months together with a decrease in the successive number of days with a relative humidity inferior to 70% during the 6 previous months (15 December 2017 to 15 June 2018 compared to 15 December 2008 to 15 June 2009).

## DISCUSSION

### Expansion of Ixodes ricinus and *Chlamydiales* in Switzerland.

The suitability index is proportional to the probability of presence of the species but involves an unknown proportionality coefficient that corresponds to the prevalence of the species. Suitability index values are thus not expected to be comparable between tick and *Chlamydiales* distribution maps. Nevertheless, distribution maps for ticks and bacteria from 2009 to 2019 highlighted an extension of the suitable areas for both species and a spread toward higher altitude. Areas suitable for Ixodes ricinus expanded from 16% to 25% of the Swiss territory, and a subsequent extension from 8% to 9.3% was observed for *Chlamydiales* bacteria. Ixodes ricinus expansion occurred all over the Swiss Plateau and toward higher altitudes in the alpine valleys and was more extended in the southwest. Newly available habitat consisted mostly of grass and forest areas. Extension of *Chlamydiales* followed similar trends but was restricted to forest areas. As Ixodes ricinus presence is favored by higher temperature, we might expect that, in the future, this expansion might continue following global warming with some limitation by dryer conditions at lower altitude.

Our results agree with the observed increased in cases of tick-borne encephalitis (TBE) in Switzerland, which spread from the eastern to the western part of Switzerland (45), leading to the extension of the vaccination recommendation (47). Similar tick expansions toward higher altitudes were observed in other European countries during the last few decades (48–50), notably in association with milder winters and extended spring and autumn seasons (51, 52).

### Variables explaining *I. ricinus* distribution.

The effective variables identified by our model are related to temperature and humidity, which reflect well the tick’s ecology. We found that a high temperature favors Ixodes ricinus, in agreement with previous studies (33, 53). However, our analysis indicated that this relationship does not hold during the driest months. This can be explained by an increased evaporation of the soil humidity under warmer temperatures, thus accentuating the desiccation risk for ticks (22). The NDVI variables, an important contribution to our model, are indicators of physiological plant activity and have often been shown to be powerful for modeling the presence of ticks because they reflect humidity conditions (22, 53). Nevertheless, our results indicated that the ambient relative humidity variables showed limited effect on the model. They may thus constitute a less precise predictor of soil humidity than the combination of NDVI variables with temperature and land cover indicators. Surprisingly, our results also showed that *I. ricinus* presence is favored by a higher percentage of artificial surfaces. This might relate to an overrepresentation of ticks collected in vegetated areas situated within urban settlements or close to roads. Indeed, we expected a sampling bias, as many tick occurrences are noted via the Tick Prevention app, in which users provide tick locations that are likely biased toward areas closer to roads or paths and thus artificial surfaces. Moreover, other tick specimens documented either by the army field campaign in 2009 or by the prospective campaign in 2018, were collected essentially in forests or close to their borders. In contrast, grassy areas, often corresponding to agricultural fields, were not sampled by the two field campaigns and were also probably less explored by the users of the application, since people are less likely to visit these areas. This might explain why our model associated a low percentage of grass vegetation as favorable for *I. ricinus* and we might have an underestimation of the suitability index in some grass areas. Nevertheless, the presence of ticks in urban and suburban areas of Switzerland has already been reported (54, 55) and the presence of vegetated areas in urban settlements or close to artificial surfaces (roads, paths, and recreational areas) may constitute favorable habitats. In addition, even if we expect some grass zones, especially at the forest border, to be highly favorable for ticks, in general pasture land, open land, and cultivated areas have been reported to be much less favorable than woodlands (22, 56, 57). Finally, in agreement with previous studies (25, 58), we observed that the morphometric parameters and the precipitation variables showed little effect on the suitability for ticks.

### Variables for *Chlamydiales* spatial distribution.

Identified effective variables for the presence of *Chlamydiales* may provide novel insights into the ecology of these bacteria. First, our results indicated that *Chlamydiales* are more likely present in ticks collected in forests or grass fields than in ticks collected close to artificial areas. The highest prevalence of *Chlamydiales* within natural areas could be explained by the presence of different hosts (likely rodents) on which ticks feed, with potentially a higher number of reservoir-competent hosts for *Chlamydiales* in natural areas. This may also relate to a higher tick abundance in natural areas, which is known to be associated with a higher prevalence of other pathogens in ticks (30) but not for all tick pathogens (55). Our results also showed that the presence of *Chlamydiales* bacteria was favored by the driest conditions (negatively associated with the number of days with a relative humidity superior to 90% and positively associated with the number of days with relative humidity inferior to 70%). Large amounts of precipitation during the coldest months also appeared to be favorable for the presence of *Chlamydiales*. Several suitable areas for *Chlamydiales* were predicted at an altitude higher than 1,000 m; thus, the highest precipitation during the coldest months could be associated with the largest snow amounts, preserving the soil from frost and leading to a higher tick survival rate (24). Finally, a shorter distance to wetlands was also highlighted as a factor favoring bacterial presence. Several *Chlamydiales* have been considered symbionts of amoebae (59), which are free-living organisms usually found at the interface between water and soil, air, or plants (59). It is therefore likely that amoebae can be found in wetlands, which might favor the transmission of *Chlamydiales* to various animal hosts on which ticks feed.

*Chlamydiales* prevalence values were heterogeneous among our data sets. In 2009, ticks were collected in forests only and *Chlamydiales* were present in 68.6% of the sites visited, with a low prevalence within pools (6.4%). A low prevalence was also observed in the ticks received from users of the Tick Prevention app in 2018 and 2019 (3.79%). In 2018, the ticks sampled during the prospective campaign were also mainly collected in forest areas and *Chlamydiales* were present in 53.7% of the sites, but with a much higher prevalence (reaching 28.13%). This rate reflects values obtained in 2010 in one specific site in the Swiss Alps (Rarogne), where a *Chlamydiales* prevalence rate of 28.1% was found in 192 pools collected in forests and meadows (60). Differences between years 2009 and 2018 could be explained by a difference in the time and sampling areas (we excluded potential PCR contaminations; see Fig. S9). As infected ticks were already present in most forest sites in 2009, spread of infection might have occurred between 2009 and 2018. Then, ticks from the Tick Prevention app were collected in sites more closely related to artificial areas, which we have shown reduces the prevalence of the bacteria.

### On the importance of considering the spatial and temporal scale of the environmental variables.

For *I. ricinus*, the most powerful models were obtained when extracting the environmental variables in a buffer with a radius of 100 or 200 m (corresponding to an area of 9 ha to 25 ha around the sampling point). This can be explained by the ecology of the species. First, the establishment of a population of ticks will probably need a suitable area that is large enough. Moreover, the presence of ticks strongly depends on the presence of hosts, which disperse across larger areas and may thus be influenced by the climatic conditions observed at some distance. Our results also indicated that a buffer radius larger than 500 m (corresponding to areas larger than 121 ha) did not improve our model. This might relate to the dispersal range of tick hosts, likely rodents, which is usually smaller (among the long dispersal hosts, the roe deer dispersal is estimated to cover around 50 and 100 ha [61]). In addition, the most powerful models were obtained when considering the climatic conditions of the 2 or 3 years preceding the sampling date. This time period appears to be relevant, as it corresponds to the estimated duration of the life cycle of ticks (22).

For the modeling of *Chlamydiales* bacteria, a small buffer (≤200 m) and a short time period (1 year or less) were favorable for some variables, whereas for some others, to consider a larger buffer (1 km or 1.5 km) and a longer time period (2 to 3 years) was better. Some variables might influence local establishment of the tick species and the ability for the bacteria to colonize and/or reproduce within it, whereas other variables may be related to the interaction of the tick with the hosts on which it feeds, which may disperse over a larger area and thus be influenced by climatic conditions at a larger scale.

Our results thus highlighted the importance of considering the environment around the sampling point for good estimation of variables in a species distribution model, while single points are commonly considered (36–44, 62). Our results also showed that the time period considered before the sampling date, with sliding windows, had a significant impact on the performance of the resulting models. This should be favored over using an average of the climatic conditions over the sampling period (36, 63) or any larger period of time (e.g., the Worldclim climatic data from 1950 to 2000 that are commonly used for species distribution modeling [64, 65]). Previous studies already suggested the use of multigrain approaches involving various spatial resolutions to consider variables affecting the presence of a species at different scales (66–68). Our study adds to the recommendation of using data based on species ecology rather than on availability (67, 69). In addition, our results showed that the temporal scale of the environmental predictors should be taken into account.

### Model performance.

Ixodes ricinus distribution models are robust, as they allowed a good discrimination between actual presence and randomly generated points and correctly predicted the presence of *I. ricinus* observed in an independent data set. *Chlamydiales* distribution models are more difficult to validate due to the limited amount of data and poor knowledge regarding their distribution. Nevertheless, our model performed relatively well for the data collected in 2018, as most of the occurrence locations had higher suitability index values than the locations where no *Chlamydiales* were identified. The year 2009 did not show such a trend, as many locations where no *Chlamydiales* were found were predicted as potentially suitable. This might be due to an absence of *Chlamydiales* colonization of these sites at the sampling time despite favorable conditions.

Our investigations considered mainly environmental factors. However, other factors, such as species interaction and species life history traits, might influence the presence of both the ticks and their bacterial pathogens (22, 29, 70–73). Also, additional abiotic factors, such as landscape fragmentation and barriers that can limit dispersal of ticks hosts (22, 72), or disturbances that can drive local populations to extinction, might play an important role (74).

The precision of our predictions is limited by the precision of the data used. The interpolated climatic grids used were produced based on weather station measurements and thus contain interpolation uncertainties that may influence the model results (70). Also, with interpolated grids, the inherent collinearity and autocorrelation may reduce the reliability of the results (58). Finally, the occurrence data are probably prone to sampling bias and do not represent a random sample of the studied population; they were collected in three separate processes, among which two constituted active surveillance while the third was passive surveillance. These elements can affect the predictions (75, 76) and, since passive surveillance can be influenced by population density, the results are likely to slightly overestimate the suitability index in urban and artificial areas compared to natural regions (75, 76).

**Conclusions**. Both Ixodes ricinus and *Chlamydiales* are a potential threat to human health, and their prevalences are currently increasing in Switzerland, with a strong expansion of ticks in forests but also into urban and suburban areas. The ticks’ expansion has already recently alarmed the Public Health Services (47), and this expansion is predicted to continue in the future due to global warming. In this context, our results offer a unique tool to identify precise locations where diseases are likely to spread through colonization of new sites and increased prevalence. The maps and associated methods developed here could thus provide critical information for decision makers to control tick-borne diseases and target prevention campaigns.

Our methodological framework allowed a coherent identification of environmental factors influencing the presence and distribution of both the Ixodes ricinus ticks and their *Chlamydiales* bacteria in Switzerland and enabled the mapping of suitability evolution across Switzerland from 2009 to 2019. Our results highlighted an important increase in suitable areas for both species and predicted their expansion toward higher altitude. Our investigations consist of an exploratory analysis of the environmental factors influencing the presence of *Chlamydiales* bacteria within ticks in Switzerland, showing an application of species distribution models to study the nested niche of a parasite within the ecological niche of its host. Finally, our study demonstrated the importance of considering the spatial and temporal scale of the environmental variables used for species distribution models.

The spread of pathogens through a vector is at the origin of major epidemics and infectious diseases and affects humans, wildlife, and agriculture. We proposed a methodological framework based on geographical systems able to provide deep insights into factors affecting patterns of disease emergence by providing a better characterization of the spatial distribution of their vectors. This method can be applied to a wide range of host-pathogen associations to identify their spread and distribution, which is expected to provide critical information for a better understanding and control of pathogens.

## MATERIALS AND METHODS

Species distribution can be modeled with various methods that use either records of presence and absence of the species or only records of presence (62, 77–79). Among them, the maximum entropy model, called Maxent (80), is a presence-only method which uses a set of georeferenced presence records and a set of environmental grid data. Based on the environmental conditions observed at locations where species were present and at background locations (i.e., random locations representative of the entire study area), Maxent uses a machine learning algorithm to estimate a suitability index value for each cell of the environmental grid, which is proportional to the probability of finding the species in that cell (81). This method has been shown to perform particularly well compared to other presence-only modeling methods, in particular based on its ability to discriminate presence sites from background locations (62, 78). We thus chose to use this model to determine the potential ecological niche of Ixodes ricinus and its associated *Chlamydiales* bacterial pathogen in Switzerland. The various steps of the method detailed in the paragraphs below are summarized in Fig. S2.

### Occurrence data for ticks and bacteria.

Data regarding tick occurrences were obtained from three different sources. First, ticks were collected by a field campaign conducted by the Swiss Army from 21 April to 13 July 2009. During this campaign, 172 forests were sampled with convenience sampling in forests of altitudes lower than 1,500 m. Ticks (62,889) were collected by flagging low vegetation using a white cloth. The ticks were then aggregated into 8,534 pools of 5 to 10 ticks (5 nymphs or 10 adults) and each pool was analyzed for the presence of *Chlamydiales* DNA by using a pan-*Chlamydiales* real-time qPCR (RT-qPCR) as described by Pilloux et al. (82), after extracting the DNA as described by Gäumann et al. (83). A pool was considered positive if the threshold cycle (*C_T_*) value was lower than 37. As a result, among the 8,534 pools, 543 were positive (6.4%) and they were located in 118 out of the 172 sampling sites (68.6%).

Second, data were obtained from the collaborative smartphone application called “Tick Prevention” (zecke-tique-tick.ch/fr/) developed by A&K Strategy GmbH, a spinoff from the Zurich University of Applied Sciences (ZHAW), in which users can indicate tick locations on a map. The application was launched in February 2015 and by the end of December 2019, there were 29,153 locations of tick observations made in Switzerland. To each observation, a spatial accuracy was assigned depending on the scale (zoomed area) to which the observation was reported by the user. For our analysis, only observations with a spatial accuracy equal to or higher than 100 m and only data collected from March to October were used. The final data set corresponded to 5,781 tick locations. Moreover, since January 2017, users bitten by a tick can send the tick removed from their body to the National Reference Centre for Tick-Transmitted Diseases (NRZK, https://www.labor-spiez.ch/en/die/bio/endiebionrz.htm). The ticks received are analyzed by three different laboratories to detect the presence of various bacteria, including *Chlamydiales*. In April 2019, 554 ticks from 506 sites were received and sequenced, among which 21 ticks (3.79%) were positive for *Chlamydiales* bacteria and were located in 19 sites (3.75%).

Finally, to increase the number of data, especially regarding *Chlamydiales* occurrences, a prospective campaign was conducted by the authors from 11 May to 24 June 2018. During this campaign, 95 sites were visited, mainly in west Switzerland. Those sites were chosen in areas predicted to be favorable for the presence of ticks based on a preanalysis of the two other data sets, as well as to maximize the environmental variability between visited sites (see Fig. S1 for more details). Whenever possible, three ticks were collected at each site by dragging a white cloth over the soil. For some sites, however, only one or two ticks could be found. Eventually, the campaign collected 256 ticks, each of which was placed in a sterile tube and kept at 4°C before being sent to the laboratory to be analyzed for the presence of *Chlamydiales* bacteria. In the laboratory, the ticks were washed once with 70% ethanol and twice with phosphate-buffered saline (PBS). DNA was extracted using the NucleoSpin DNA insect kit (Macherey-Nagel) with NucleoSpin bead tubes type E and MN bead tube holder in combination with the Vortex-Genie 2. The manufacturer’s protocol was slightly adapted by performing disruption for 20 min followed by 2 h of incubation at 56°C in order to allow proteinase K digestion. DNA was then analyzed using the pan-*Chlamydiales* qPCR developed by Lienard et al. (84). A tick was considered positive for the presence of *Chlamydiales* if either of the two replicates was positive or if one of the two was highly positive (*C_T_* value < 35). As a result, 72 out of the 256 ticks were positive (28.13%) in 51 out of 95 sites (53.6%).

The characteristics of each data set are summarized in Table 1.

**TABLE 1:** Characteristics of the three data sources with respect to Ixodes ricinus occurrence and infection by *Chlamydiales* bacteria^*a*^

Parameter	Swiss Army field campaign	“Tick Prevention” app ticks recorded	“Tick Prevention” app ticks sent for analysis	Authors' prospective campaign
Observation/sampling dates	21 April 2009–13 July 2009	09 March 2015–30 Oct 2019	04 April 2017–07 April 2019	11 May 2018–24 June 2018
No. of sites	172	5,781	506	95
No. of individual ticks	62,889	5,781	554	256
No. of adults	20,313	-	58	114
No. of nymphs	42,576	-	444	142
No. of larvae	0	-	50	0
No. of pools	8,534	/	/	/
No. of ticks/pools infected	543	-	21	72
Infection rate in ticks/pools (%)	6.34	-	3.79	28.13
No. of sites infected	118	-	19	51
Infection rate in sites (%)	68.6	-	3.75	53.68

a The data obtained via the Tick Prevention app are divided into two data sets (columns 3 and 4). The first data set (column 3) corresponds to tick locations recorded on the app and includes a majority of ticks for which no information regarding *Chlamydiales* bacteria was available. This data set was used in the modeling of the distribution of Ixodes ricinus only. The second data set (column 4, which represents a subset of the data set in column 3) contains some ticks that were sent for the laboratory analysis of *Chlamydiales*. This data set was therefore used in the modeling of *Chlamydiales* distribution. Data from the two other sources (columns 2 and 5) were used both for the modeling of *I. ricinus* and *Chlamydiales*. -, no information; /, not applicable.

### Environmental data.

To characterize the environmental conditions potentially influencing the spatial distribution of Ixodes ricinus and *Chlamydiales*, several types of information were retrieved for the whole territory of Switzerland regarding (i) the morphometry, (ii) the land cover, and (iii) the climate.

To characterize the morphometry of each data point site, seven indicators were derived from the digital elevation model provided by USGS/NASA SRTM data version 4.1, at a 90-m resolution (85). The chosen indicators were computed using the SAGA GIS 2.3.2 software (86) and represent slope, aspect, general curvature, morphometric protection index, terrain ruggedness, sky-view factor, and topographic wetness. The definition of each of these indicators and the exact procedure followed to derive them are detailed in File S3 in the supplemental material.

To characterize the land cover, we first used the land cover statistics from the Swiss Federal Statistical Office (OFS) (87). From this data set, we retrieved the classification of each Swiss hectare into six land cover types representative of the period 2004 to 2009 as follows: artificial areas, grass and herb vegetation, brush vegetation, tree vegetation, bare land, and watery areas. To better classify the forest type, we computed in R (88) the percentage of coniferous trees in each forest based on a data set provided by the OFS at a 25-m resolution, which classifies the forests of Switzerland into four classes as follows: pure coniferous, mixed coniferous, mixed broadleaved, and pure broadleaved (89). Second, we retrieved the vector landscape model swissTLM3D 2016 from the Swiss Federal Office of Topography (90) and we used the function “Proximity” in the QGIS 2.14.7 software (91) to derive four indices characterizing the minimal Euclidean distance to watery areas as follows: distance to wetland, to watercourses, to stagnant water, and to any watery elements. Third, we retrieved the 16-day composite normalized difference vegetation index (NDVI) available in the MODIS satellite products at a 250-m resolution (92), from which we derived in R the average, minimum, maximum, and range of monthly mean NDVI. More details regarding all those land cover data and the derived indicators are available in Fig. S3.

Finally, several indicators were computed to summarize the climatic conditions of each data point site. They were derived from monthly temperature (average, minimal, and maximal) and sum of precipitation grids computed at a 100-m resolution by the Swiss Federal Institute for Forest, Snow and Landscape Research (www.wsl.ch), based on data from MeteoSwiss (www.meteoswiss.ch) and using the Daymet software (93). From these data, 31 indicators were derived to represent the climatic conditions during the period of interest and before the sampling date (from 1 to 36 months preceding the sampling date; see the “Data extraction” section for more details). These indicators are presented in Fig. S3 and they summarize (i) the values of the monthly mean, minimal, and maximal temperature and sum of precipitation (8 indicators); (ii) the variation of monthly temperature and precipitation (5 indicators); (iii) the temperature of the warmest and coldest months (2 indicators); and (iv) the temperature and precipitation of the three consecutive warmest, coldest, wettest, and driest months (16 indicators). In addition, grids of the daily maximum and minimum temperature values at a 1-km resolution were obtained from MeteoSwiss. From these data sets, we estimated the daily saturated and ambient vapor pressure using the Tetens formula (94) and by approximating the temperature at dew point by the minimum temperature (95). We used them to compute the daily relative humidity and to derive 22 indicators summarizing the monthly (9 indicators) and daily (13 indicators) values of relative humidity. All these climatic predictors were computed in R, with the detailed procedure presented in Fig. S3. In total, this resulted in 77 environmental indicators, each of which was resampled to a final spatial resolution of 100 m.

### Data extraction.

The values of the 77 environmental predictors were extracted for each data point site (tick occurrence) according to their coordinates using the function “extract” from the R “raster” package. The climatic and NDVI variables were retrieved as a function of the sampling dates. To assess the influence of the conditions before sampling, we retrieved these variables for 1 month, 3 months, 6 months, 1 year, 2 years, and 3 years before the sampling date. For the other stable predictors such as morphometric predictors, land cover type, percentage of coniferous cover in forest, and distances to watery areas one single extraction was used for all sampling dates over the period of analysis (from 2009 to 2019).

To assess the influence of the environmental conditions surrounding the sampling points, for each environmental predictor we also computed the mean value observed in square buffers centered on the sampling point, with radii of 100 m, 200 m, 500 m, 700 m, 1 km, and 1.5 km. Raster layers were also computed for each of these indicators, with every buffer radius and time period for the month of June from 2009 to 2019. For each pixel, the computation of mean values considering a square buffer around the pixel was done with a moving-window procedure implemented in R, based on the “focal” function from the “raster” package.

Finally, we also extracted all predictors for a generated background data set composed of sites with 10,000 coordinates randomly localized in Switzerland. As with the presence records, we also computed the mean values in buffers and considered different time periods for the extraction of NDVI and climatic variables. To this end, a fictive sampling date was assigned to each background location, which was randomly selected from the distribution of observed sampling dates on the presence records (Fig. S4).

### Ixodes ricinus modeling.

**(i) Selection of environmental variables.** In order to compare the influences of the time period and buffer radius on the performance of the model, independent Maxent species distribution models were derived using environmental predictors extracted successively for each combination of buffer radius (100 m, 200 m, 500 m, 700 m, 1 km, and 1.5 km) and time period (1 month, 3 months, 6 months, 1 year, 2 years, and 3 years), i.e., one Maxent model was derived using all environmental conditions extracted within a 100-m buffer and 1 month preceding the sampling date, then a second model was derived using 200-m buffer and 1 month, etc. In addition, to determine if the performance of the model could be increased by selecting a different buffer radius and time period for the different environmental variables, we computed a “combination model” in which we selected the most significant combination of buffer radius and time period individually for each environmental variable. To this end, we performed a Student’s *t* test to identify, for each environmental variable, the combination that best discriminates the tick’s presence from background locations. The computation was done using the function “t.test” in R and the discriminative power of variables was considered significant if the *P* value of the *t* test was lower than 0.01 after a Bonferroni correction for multiple comparisons. For each environmental variable, we then kept only the combination of buffer radius and time period showing the highest *t* value. The “combination” model was then derived using this “combination” set of variables.

As some environmental variables considered might be correlated, we used two methods to preselect uncorrelated environmental predictors. In the first one, we ran a principal component analysis (PCA) on the variables to retrieve independent components. The coordinates of the PCA components were then used as environmental predictors to run the species distribution model. In the second method, for each pair of variables showing a Pearson correlation higher than 0.8, we kept only the variable with the highest *t* value in the *t* test previously computed. In addition, to avoid multicollinearity between our variables, which can influence the resulting models, we computed the variance inflation factor (VIF) for each variable using the R function “vif.” This index estimates how much the variance of a regression coefficient is inflated due to collinearity, and VIF values higher than 10 can be considered indicative of problematic collinearity (96). We thus successively removed the variable showing the strongest VIF index values, until the highest VIF value was lower than 10. Only the remaining variables were used to train the model.

**(ii) Maxent modeling.** Species distribution modeling was performed using the Maxent algorithm (80) implemented in the R package “maxnet” (97). Maxent estimates a suitability index which is proportional to the probability of the presence of the species knowing the environmental conditions of a site of interest (81). The computation requires the values of environmental predictors observed on sites where presence was recorded and on background locations (i.e., locations representative of the entire study area). The model was trained with all Ixodes ricinus occurrences available for years 2009 to 2017 and the occurrences from the 2018 prospective campaign. This represents a total of 2,293 presence points. The occurrences reported by the users of the Tick Prevention app in 2018 and 2019, with 3,751 presence points, were kept as an independent data set used to test the models.

Since the performance of the Maxent models is known to be notably influenced by the background point selection, environmental variable selection, feature types, and regularization parameters (75, 98–100), we tested different alternatives regarding them. For the selection of background points, we tested two options: we either (i) used the 10,000 points randomly selected in the Swiss territory or (ii) used only the random points situated below 1,500 m in altitude, where tick occurrence is more likely. For the environmental variables, we used the two procedures to derive uncorrelated set of variables, i.e., the coordinates of the PCA components or the variables filtered by the previously described method based on Pearson correlation and variance inflation factor. Moreover, when using the PCA components, we considered either all components of the PCA or only the components needed to retain 50%, 70%, 80%, 90%, or 95% of the variance. For the feature types, we tested the use of linear features only, or the combination of linear and product, linear and quadratic, or linear, product, and quadratic together. Finally, we varied the regularization constant parameter, which is used to select against complex models that are unlikely to generalize well, with constant values equal to 1, 2, 5, or 10 (the higher the value, the stronger the penalization).

In order to perform a cross-validation procedure, we used 75% of the occurrences and background points to train the model and kept 25% to test it. The training and testing occurrences were selected randomly and 20 different runs were computed. All models were projected using the “cloglog” scaled output (101), interpreted in terms of suitability index to avoid making assumptions regarding the prevalence of the species.

**(iii) Model evaluation.** The models were compared based on four criteria. First, the area under the receiver operating curve (AUC) (102) was computed on the testing data set. The mean value of AUC_test_ over the 20 runs was used as a measure of discrimination power. The AUC is a measure commonly used for the evaluation of species distribution models (62, 103). It has the advantage of being threshold-independent but needs to be used in combination with other evaluation parameters (104–106). Therefore, we used as a second evaluation measure the omission error rate, which reflects the accuracy of the model. The computation of this rate requires the definition of a threshold value to classify the predictions into binary presences or absences. Based on the receiver operating curve, we chose the threshold which maximizes the sum of specificity and sensitivity and therefore minimizes the misclassification rate (107). Omission errors were computed on both the testing and independent (3,751 points from 2018 and 2019) data sets. Finally, to avoid the selection of complex models, which would be difficult to interpret and probably prone to overfitting, we used a third evaluation measure that selected against models having high number of coefficients (following the principle of information criterion) (108).

To combine the four evaluation parameters and select the most powerful model, we assigned four performance ranks to each model as a function of each evaluating parameter and we selected the model which minimizes the sum of ranks. We then applied the best model to the raster layers to map the predicted suitability across all of Switzerland for the months of June from 2009 to 2019.

**(iv) Identification of effective variables.** In order to identify the environmental variables contributing the most to the model, we implemented in R a jackknife procedure as proposed by Phillips (101). For each environmental predictor, we computed the Maxent model with only this variable and calculated the corresponding AUC (AUC_only_). Variables leading to high values of AUC_only_ therefore contribute a lot to the model by themselves. Similarly, we successively computed models with all variables except the one of interest and we computed the corresponding AUC_without_. Predictors associated with high values of AUC_without_ were identified as containing important information that is not present in the other variables.

### *Chlamydiales* modeling.

**(i) Background data set.** To model the distribution of *Chlamydiales* bacteria within ticks, we used a similar procedure to that used for Ixodes ricinus. The modeling was also done using Maxent, based on the 186 occurrence points available for 2009 and 2018. As for *I. ricinus*, the modeling required the definition of background data. Since we are interested in the probability of finding *Chlamydiales* within ticks, background points have to represent the environmental conditions of the ecological niche for the tick. Consequently, we built a background data set in two steps. First, we selected the points where ticks not infected by *Chlamydiales* have been observed (374 points). Second, in order to avoid a model discriminating presences from background due to differences in sampling dates, we completed the background data set such as to have a distribution of sampling months and sampling years similar to that in the presence data set (Fig. S4). This was achieved by selecting random points within areas predicted to be suitable for ticks, based on the suitability predicted by the models previously derived for Ixodes ricinus. The final background data set contained 1,028 data points.

**(ii) Variable selection and modeling.** The same procedure as for the modeling of the tick suitability was then applied: (i) computation of a *t* test to select a “combination” data set of environmental variables; (ii) selection of uncorrelated variables with either a PCA or a correlation/VIF procedure; and (iii) running of Maxent models by testing various parameters (methods to select uncorrelated variables, feature types, and regularization parameters). In order to build models for the suitability of *Chlamydiales* within areas suitable for ticks, the predicted suitability for *Chlamydiales* obtained by the Maxent model was then multiplied by the suitability obtained for *I. ricinus*.

As for *I. ricinus*, 20 runs were computed for each model, using 75% of the data to train the model and 25% to test it. The ranking procedure used to evaluate the models was slightly different from the one used for the tick. The AUC_test_ and the number of coefficients were used similarly, but the omission rates on testing and independent data sets were replaced by two other indicators: (i) the difference between the mean of suitability values predicted on occurrences sites in 2009 and the mean suitability predicted on sites without *Chlamydiales* in 2009; and (ii) the same difference for 2018. Indeed, even if sites where no *Chlamydiales* were found could not be considered proper absences, we suspected the probability of finding *Chlamydiales* to be lower on these sites. A model showing a lower suitability in areas where *Chlamydiales* were not identified compared to occurrence sites would therefore be considered more powerful.

### Data availability.

The main R codes developed for this study are available on GitHub (https://github.com/estellerochat/SDM-Chlamydiales). Ticks and *Chlamydiales* occurrence data from the Swiss Army field campaign and the prospective campaign have been submitted to Zenodo (doi:10.5281/zenodo.4028822). The corresponding extractions of environmental predictors considering various buffers and time periods are available in the same repository. Tick occurrences from the participating smartphone application Zecke-tique-tick should be requested from their owner, A&K Strategy GmbH, using the contact form available at https://zecke-tique-tick.ch/fr/contact-et-infos/. A&K Strategy GmbH will then specify the terms of use through material transfer agreements.

## Supplementary Material

Supplemental file 1
