# Supplementary material for: Nested Species Distribution Models of Chlamydiales in Ixodes ricinus (Tick) Hosts in Switzerland
Source: Appl Environ Microbiol. 2020 Dec 17;87(1):e01237-20. doi: 10.1128/AEM.01237-20 (PMC7755253; doi:10.1128/AEM.01237-20)
Supplement: Supplemental file 1 [file AEM.01237-20-s0001.pdf]

# Supplemental Material

## Nested species distribution models of *Chlamydiales* in tick host *Ixodes ricinus* in Switzerland

Estelle Rochat, Séverine Vuilleumier, Sebastien Aeby, Gilbert Greub, Stéphane Joost

## Supp. File 1 – Prospective campaign

In order to select the sites visited during the prospective campaign, we proceeded in four steps:

1. Based on the already available ticks occurrences (data from the Swiss army field campaign and data from the smartphone application for 2015 to 2017), we run a MAXENT model to obtain a first map of suitability for ticks.
2. We performed a PCA on the environmental predictors extracted in the pixels predicted as potentially suitable for ticks (suitability from step 1 greater than 0.2).
3. We computed a k-means classification on the components of the PCA. This allowed us to define 5 environmental clusters on the Swiss territory (Figure 1).
4. We manually selected the sampling sites:
    - in areas defined as potentially suitable for ticks (suitability predicted at step 1 greater than 0.2),
    - such as to sample sites in each environmental cluster defined at step 2,
    - such as to maximise the number of sites that can be visited during one day (i.e. the sites can be link together by roads or paths),
    - so as to complete the dataset already available regarding the presence of *Chlamydiales* bacteria (data from the Swiss army field campaign).

As a result, 96 sites were visited and ticks were found in 81 of them, corresponding to 228 ticks. In addition, some relatives of the authors provided ticks they had collected. By this way, 28 additional ticks were received, from 14 new sites. In total, the prospective campaign therefore provided 256 ticks from 95 sites.

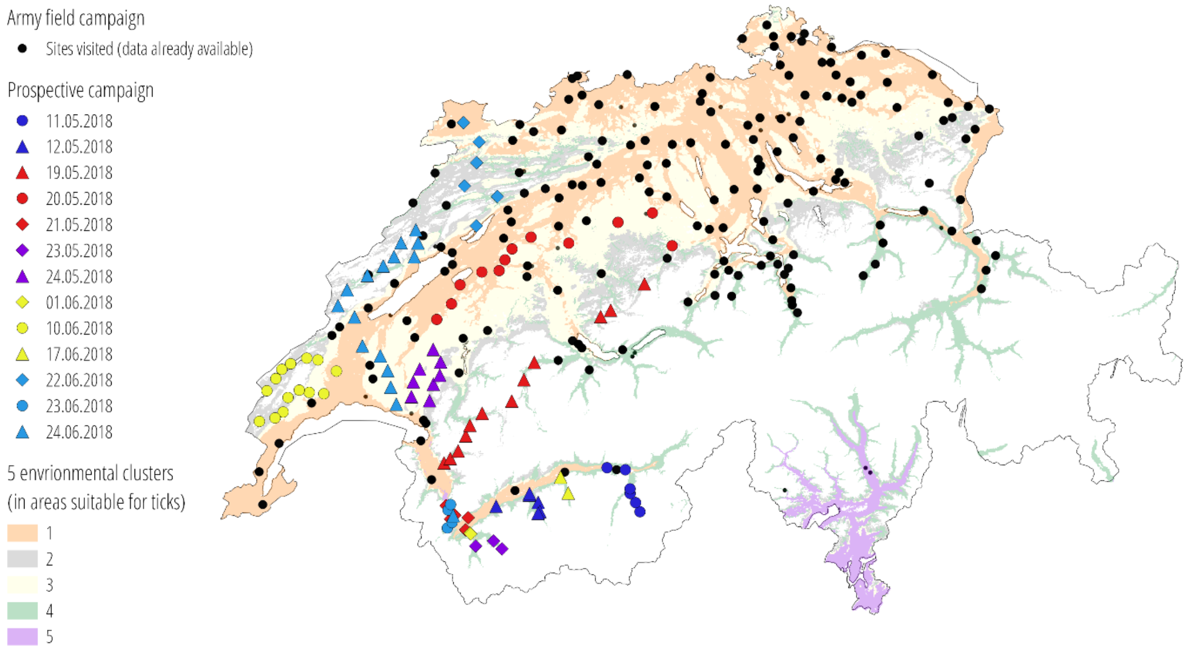


**Figure 1**: Map of the environmental clusters defined with the k-means performed on the PCA-components of the environmental predictors, and location of the sites visited during the prospective campaign.

## Supp. File 2 – Method

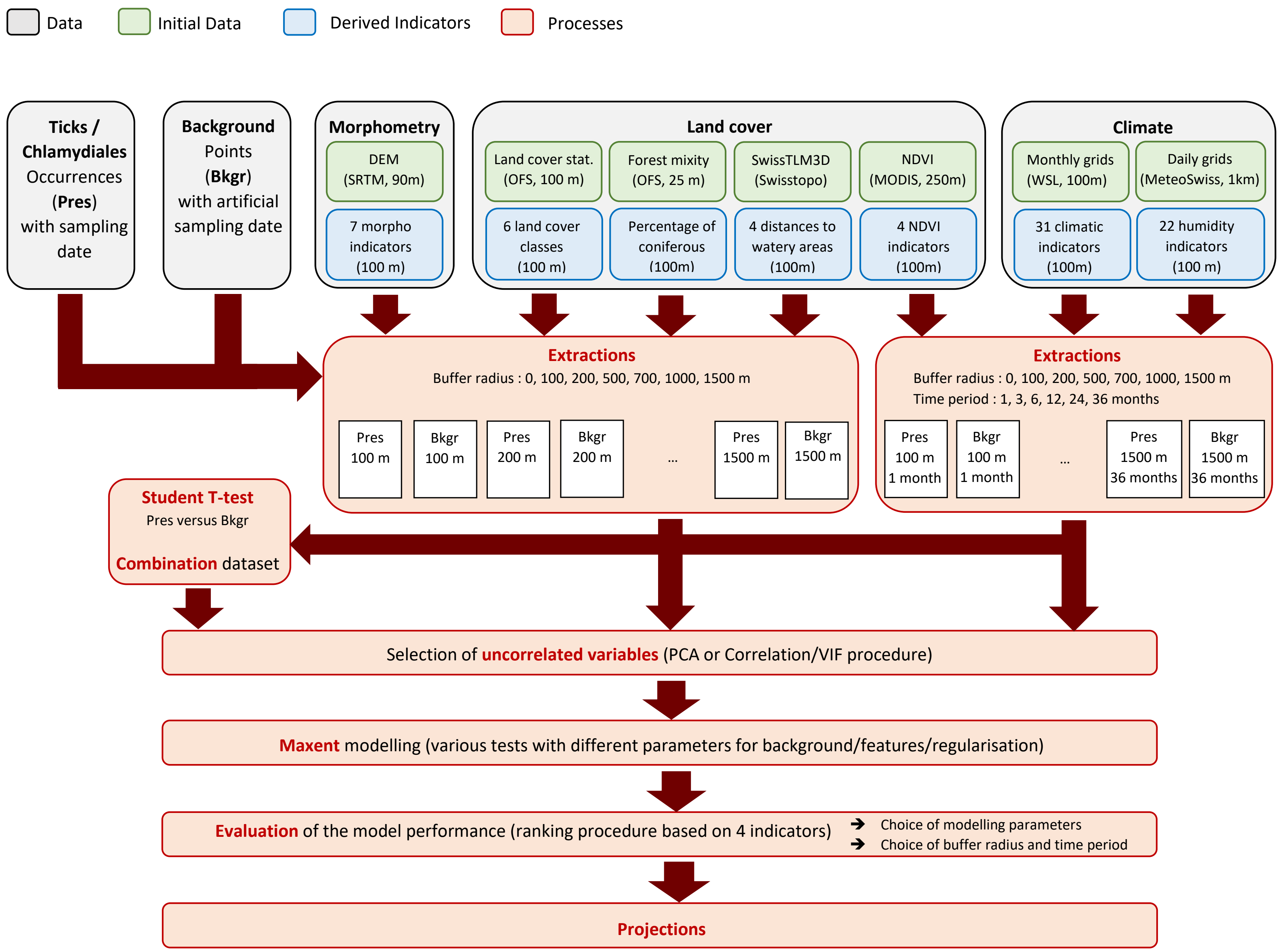

# Supp. File 3 – Environmental data

## Morphometry

Initial data
- SRTM Digital Elevation Model
- **Spatial resolution**: 90 m
- **Source**: NASA Shuttle Radar Topography Mission (https://www2.jpl.nasa.gov/srtm/), with hole-filled version processed by the CIAT Agroecosystems Resilience project (Jarvis, 2008)
- **URL:** http://srtm.csi.cgiar.org/

Pre-processing
- The two tiles covering Switzerland were downloaded and merged using the QGIS 2.14.7 software (function merge from GDAL).
- The merged dataset was then resampled at a 100-m resolution and cropped to the Swiss extent using the SAGA "resampling" tool accessed from QGIS 2.14.7 and using the interpolation method: mean value (cell-area weighted).
- Null values were assigned for all pixels outside the Swiss borders.

Slope, Aspect, General Curvature (GC)
- **Method**: These indicators were computed in SAGA GIS 2.3. using the tool "Terrain Analysis > Morphometry > Slope, Aspect, Curvature", with the method "9 parameter 2nd order polynom" (Thorne *et al.*, 1987) and the units defined in degrees.

Morphometric Protection Index (MPI)
- **Definition**: This indicator provides a dimensionless index expressing how well an area is protected from the surrounding relief, based on the analysis of the environment surrounding each pixel up to a given distance. It is equivalent to the positive openness described by Yokoyama et al. (2002).
- **Method**: This indicator was computed in SAGA GIS 2.3.2 using the tool "Terrain Analysis > Morphometry > Morphometric Protection Index and the default parameters (the relief in the surrounding 2km of each pixel is taken into account).

Terrain Ruggedness Index (RI)
- **Definition**: This indicator compares the elevation of one pixel with the elevation of the neighbouring pixels to provide a measure of terrain heterogeneity (Riley *et al.*, 1999).
- **Method**: This indicator was computed in SAGA GIS 2.3.2 using the tool "Terrain Analysis > Morphometry > Terrain Ruggedness Index" with the default parameters (Radius (Cells)=1 indicating that one neighbour cell is considered in each direction).

Sky-view factor (SVF)
- **Definition**: This indicator provides an indication of the portion of sky that is obstructed by the surrounding relief: 0 = completely obstructed, 1=completely visible (Böhner and Antonić, 2009, p. 8)
- **Method**: This indicator was computed in SAGA GIS 2.3.2 using the tool "Terrain Analysis > Lighting, Visibility > Sky view factor" with the default parameters (Maximum search radius = 10 km).

Topographic Wetness Index (TWI)

- **Definition**: This indicator is defined from the ratio of the catchment area (area draining water to a given cell) to the local slope (indicator of the capacity to evacuate the water received) and is used as a proxy of soil moisture (Kopecký and Čížková, 2010).
- **Method**: First we computed the specific catchment area in SAGA GIS 2.3.2 using the tool "Terrain Analysis > Hydrology > Flow Width and Specific Catchment Area" with the default parameters (Aspect method). The TWI was then computed in SAGA GIS 2.3.2 using the tool "Terrain Analysis > Hydrology > Topographic Wetness Index" with the default parameters (Standard method).

## Land Cover

Land cover classification

- Land cover classification in 6 classes : artificial areas, grass and herb vegetation, brush vegetation, tree vegetation, bare land and watery areas
- **Spatial resolution** : 100 m
- **Source**: Swiss Federal Statistical Office (OFS, 2017)
- **URL:** https://www.bfs.admin.ch/bfs/fr/home/statistiques/espace-environnement/nomenclatures/arealstatistik/nolc2004.html
- **Processing:** the only processing was to rasterise the data using the function rasterise in QGIS 2.14.7 (the initial data was available as a .csv file)

Percentage of coniferous in forest

**Initial Data**

- Raster file classifying the forests of Switzerland into four classes: pure coniferous, mixed coniferous, mixed broadleaved and pure broadleaved.
- **Spatial resolution**: 25 m, but with a grid translated by 12.5m as compared to the other data.
- **Source**: Swiss Federal Statistical Office (OFS, 2013)
- **URL:** https://www.bfs.admin.ch/bfs/fr/home/services/geostat/geodonnees-statistique-federale/sol-utilisation-couverture/donnees-derivees-autres-donnees/mixite-forets.html

**Processing**

- First, the raster with a spatial resolution of 25m was resampled in QGIS 2.14.7 to a raster with a spatial resolution of 12.5 m using the function "resample" with the nearest neighbour method.
- A percentage of conifers was then assigned to each 12.5m pixel according to the classification proposed by OFS:
    - 0 = no-forest => 0 % coniferous
    - 1 = coniferous forest => considered 100% coniferous
    - 2 = mixed forest predominantly coniferous => considered 70% coniferous
    - 3 = mixed forest predominantly broadleaved => considered 30% coniferous
    - 4 = broadleaved forest => considered 0% coniferous
    - 9 = unclassified => considered no forest => 0% coniferous
- The 12.5 m raster was aggregated to a 100 m target grid, by computing for each target cell the average percentage of coniferous using the tool "zonal statistics" in QGIS 2.14.7.
- The resulting grid was rasterised using the "rasterise" function in QGIS 2.14.7.

Distances to water areas

**Initial Data**

- Vector landscape model SwissTLM3D from 2016
- **Source**: Swiss Federal Office of Topography (O'Sullivan *et al.*, 2008)
- **URL:** https://shop.swisstopo.admin.ch/en/products/landscape/tlm3D

**Processing**

- All the elements characterising watery areas were extracted from the landscape model
    - For running water: the lines "Fliessgewaesser" and the polygons "Fliessgewaesser" extracted from the LandCover (Bodenbedeckung) polygons
    - For stagnant water: the lines "Stehendes Gewasser" and the polygons " "Stehendes Gewasser" extracted from the land cover (Bodenbedeckung) polygons
    - For the wetlands: the polygons "Feuchtgebiet" extracted from the land cover (Bodenbedeckung) polygons
- The vector layers were rasterised using the "rasterise" function in QGIS 2.14.7.
- For each pixel, the minimal Euclidean distance to each water category was then computed using the function "Raster > Analysis > Proximity" in QGIS 2.14.7. This resulted in three raster layers, representing the minimum distance to running water elements, stagnant water and wetlands, respectively.
- Finally, the minimum of the three raster files was used as the minimal distance to any watery element.

Vegetation Indexes

**Initial Data**

- MODIS Terra 16-days composite NDVI
- **Definition**: The 16-day composite NDVI is produced on 16-day intervals and provide an indicator of the greenness of the vegetation during these 16 days. NDVI is derived from the reflectance in the red and near-infrared (NIR) bands obtained from the images of the MODIS satellite.

$$NDVI = \frac{NIR - Red}{NIR + Red}$$

A large amount of red wavelengths are absorbed by the vegetation during photosynthesis, while the near infrared is reflected, in a proportion that depends in particular on the leaf area index. Land covered by vegetation will therefore show a large difference between NIR and red reflectance, resulting in high NDVI values.

- **Units**: The valid range of value is -2000 to 10'000 with a scale factor of 0.0001 (i.e. a value of -2000 correspond to a NDVI of -0.2, whereas a value of 10'000 indicates a NDVI equals to 1.0)
- **Spatial resolution**: 250 m
- **Source**: NASA Moderate Resolution Imaging Spectoradiometer (MODIS) (Huete *et al.*, 1999)
- **URL:** https://modis.gsfc.nasa.gov/data/dataprod/mod13.php
- **Download:** https://search.earthdata.nasa.gov/

**Processing**

- We downloaded all images for the years 2006 to 2019.
- The hdf4 files were converted to .tif format using the "gdal_translate" function in R (R Development Core Team, 2008)
- The MODIS data being in sinusoidal projection, rasters were reprojected in the CH1903/LV03 projection system using the "projectRaster" function of the "raster" package in R

- The files were cropped and resampled to a 100m resolution using the “crop” and “resample” function from the “raster” package in R
- For each pixel, the monthly mean values were then computed in R.
- Finally, remaining null values were replaced by the average value of the neighbouring pixels using the “focal” function from the “raster” package in R.

**Derived variables**

Four indicators were derived for the period of interest (1, 3, 12, 24 or 36 months before the sampling date).

*1.* Average of monthly mean NDVI (***meanNDVIm***)
*2.* Minimum of monthly mean NDVI (***minNDVIm***)
*3.* Maximum of monthly mean NDVI (***maxNDVIm***)
*4.* Range of monthly mean NDVI (***RgeNDVIm***)

## Climate

<u>Temperature and precipitation</u>

**Initial Data**

- Monthly mean, maximum and minimum temperature and monthly sum of precipitation
- **Spatial resolution**: 100 m
- **Source**: grids computed by the Swiss Federal Institute for Forest, Snow and Landscape Research (WSL), based on data from MeteoSwiss weather stations and a 100 m resolution digital elevation model aggregated from the DHM25 of SwissTopo. The computation was performed using Daymet software (Thornton *et al.,* 1997) and the reported mean absolute error (crossvalidation) is ~1°C for temperature and ~10-15% for precipitation (personal communication from WSL).
- **URL:** https://www.wsl.ch/de/projekte/climate-data-portal.html
  DHM25 Swisstopo**:** https://shop.swisstopo.admin.ch/fr/products/height_models/dhm25
  MeteoSwiss: https://www.meteoswiss.admin.ch/home/measurement-values.html

**Derived variables**

First, 15 indicators were derived for the period of interest (1, 3, 12, 24 or 36 months before the sampling date). Some of these indicators are very close to the worldclim bioclimatic variables (https://worldclim.org/data/bioclim.html). They were computed in R using two custom R functions (one defined for the treatment of data frame and the other for raster layers). The two functions are available in: https://github.com/estellerochat/SDM-Chlamydiales.

1. Average of the monthly mean temperatures over the period of interest (***meantmean***)
2. Maximum of the monthly maximal temperatures over the period of interest (***maxtmax***)
3. Minimum of the monthly maximal temperatures over the period of interest (***mintmax***)
4. Maximum of the monthly minimal temperature over the period of interest (***maxtmin***)
5. Minimum of the monthly minimal temperatures over the period of interest (***mintmin***)
6. Average of the monthly range of temperatures (***meanMoRge***)
7. Global range of temperature (maxtmax-mintmin) (***tRge***)
8. Isothermality (100*meanMoRge / tRge) (***isotherm***)
9. Temperature seasonality (standard deviation*100) (***tseason***)
10. Mean temperature of the coldest month (***mintmean***)
11. Mean temperature of the warmest month (***maxtmean***)
12. Total sum of precipitation (***sumprec***)
13. Maximum of the monthly sums of precipitation over the period of interest (***maxprec***)
14. Minimum of the monthly sums of precipitation over the period of interest (***minprec***)
15. Precipitation seasonality (Coefficient of Variation CV = sd/mean*100) (***pseason***)

Secondly, 16 additional indicators were derived when the period of interest was exceeding 3 months (i.e. 6, 12, 24 or 36 months) (CM=”consecutive months”)

1. Average of the monthly mean temperature of the 3 coldest CM (***meantmean3cold***)
2. Average of the monthly minimal temperature of the 3 coldest CM (***meantmin3cold***)
3. Average of the monthly maximal temperature of the 3 coldest CM (***meantmax3cold***)
4. Sum of precipitation of the 3 coldest CM (***prec3cold***)
5. Average of the monthly mean temperature of the 3 warmest CM (***meantmean3warm***)
6. Average of the monthly minimal temperature of the 3 warmest CM (***meantmin3warm***)
7. Average of the monthly maximal temperature of the 3 warmest CM (***meantmax3warm***)
8. Sum of precipitation of the 3 warmest CM (***prec3warm***)
9. Average of the monthly mean temperature of the 3 wettest CM (***meantmean3wet***)
10. Average of the monthly minimal temperature of the 3 wettest CM (***meantmin3wet***)
11. Average of the monthly maximal temperature of the 3 wettest CM (***meantmax3warm***)
12. Sum of precipitation of the 3 wettest CM (***prec3wet***)
13. Average of the monthly mean temperature of the 3 driest CM (***meantmean3dry***)
14. Average of the monthly minimal temperature of the 3 driest CM (***meantmin3dry***)
15. Average of the monthly maximal temperature of the 3 driest CM (***meantmax3dry***)
16. Sum of precipitation of the 3 driest CM (***meantmean3dry***)

Humidity variables

**Initial Data**

- Daily mean, maximum and minimum temperature
- **Spatial resolution**: 1 km
- **Source**: MeteoSwiss
- **URL:** https://www.meteoswiss.admin.ch/home/climate/swiss-climate-in-detail/raeumliche-klimaanalysen.html

**Processing**

- The daily grids were imported in R
- The daily relative humidity was computed using the same procedure as in Zimmermann *et al.* (2001)
    - Compute the average daytime temperature following Running *et al.* (1987)

$$t_{day} = 0.394\ t_{min} + 0.606\ t_{max}$$

    - Compute ambient vapour pressure using the Tetens equation for temperatures above 0°C (Murray, 1966) and minimum temperature as an approximation of dew point temperature (Running *et al.*, 1987)

$$VP_{amb} = 610.78\ \exp\left(\frac{17.269\ t_{min}}{237.3 +\ t_{min}}\right)$$

    - Compute the potential vapour pressure of saturated air for daytime temperature using the Tetens equation for temperatures above 0°C (Murray, 1966) and the previously computed average daytime temperature:

$$VP_{sat} = 610.78\ exp\left(\frac{17.269\ t_{day}}{237.3 +\ t_{day}}\right)$$

    - Compute the relative Humidity (in %)

$$RH = \frac{VP_{amb}}{VP_{sat}} * 100$$

- The daily relative humidity grids were then aggregated to compute four monthly grids:

1. Monthly mean of RH
2. Monthly median of RH
3. Monthly quantile 0.25 of RH
4. Monthly quantile 0.75 of RH

**Derived variables**
22 indicators were derived for the period of interest (1, 3, 12, 24 or 36 months before sampling date). They were computed in R using two custom R functions (one defined for the treatment of data frame and the other for raster layers). The two functions are available in: https://github.com/estellerochat/SDM-Chlamydiales.

1. Average of monthly mean RH (***meanRHmean***)
2. Average of monthly median RH (***meanRHq050***)
3. Minimum of monthly mean RH (***minRHmean***)
4. Maximum of monthly mean RH (***maxRHmean***)
5. Minimum of monthly 0.25 quantile of RH (***minRHq025***)
6. Minimum of monthly 0.75 quantile of RH (***minRHq075***)
7. Maximum of monthly 0.75 quantile of RH (***maxRHq075*)**
8. Range of monthly RH (***RHrge***)
9. Average of the monthly ranges of RH (***RHMoRge***)
10. Mean daily RH (***meanRHD*)**
11. Median daily RH (***medRHD*)**
12. Minimum daily RH (***minRHD***)
13. Maximum daily RH (***maxRHD*)**
14. Range of daily RH (***rangeRHD***)
15. Quantile 0.25 of daily RH (***q025RHD***)
16. Quantile 0.75 of daily RH(***q075RHD***)
17. Number of days with RH<70% (***ndRHDinf70***)
18. Number of days with RH<80% (***ndRHDinf80***)
19. Number of days with RH>90% (***ndRHDsup90***)
20. Maximum number of consecutive days with RH< 70% (***ncdRHDinf70***)
21. Maximum number of consecutive days with RH< 80% (***ncdRHDinf80***)
22. Maximum number of consecutive days with RH>90% (***ncdRHDsup90***)

# Supp. File 4 - Background datasets

## *Ixodes ricinus*

<u>Sampling date</u>

Distribution of sampling dates (month and year) of the occurrence dataset (presences, 2293 points) and selected background points below 1500 m (6050 points).

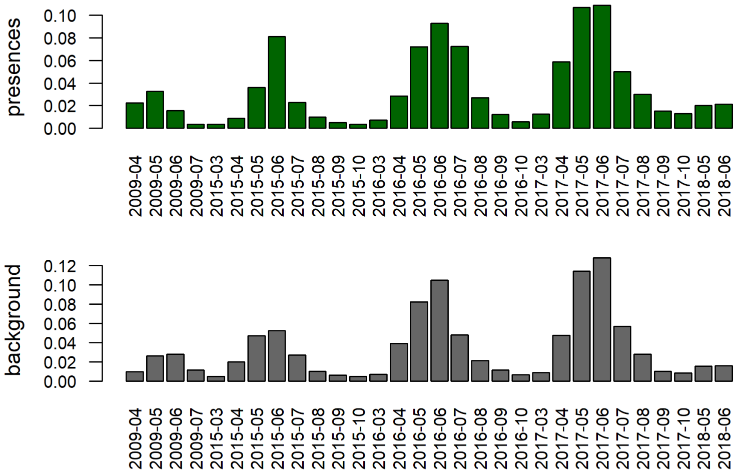


<u>Altitude</u>

Distribution of altitude values of the occurrence dataset (presences, 2293 points) selected background points below 1500 m (6050 points).

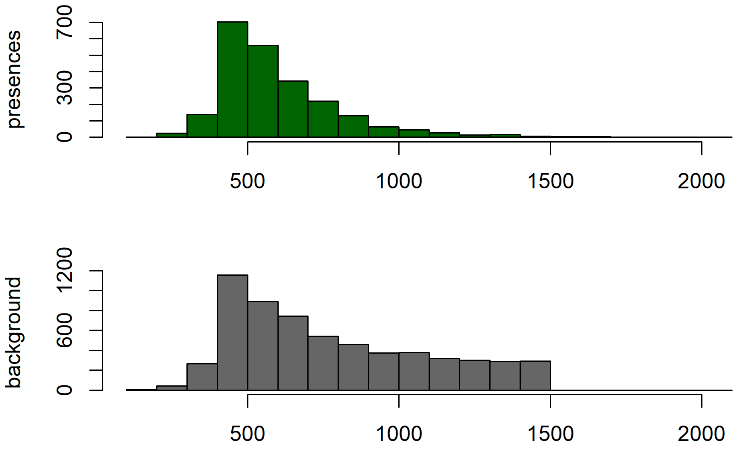

## *Chlamydiales*

### Sampling date

Distribution of sampling dates (month and year) of the occurrence dataset (presences, 186 points) and background points (1029 points).

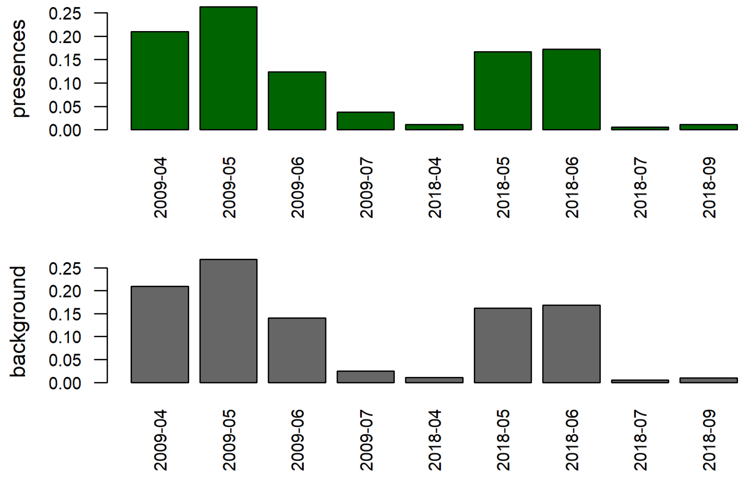


### Altitude

Distribution of altitude values of the occurrence dataset (presences, 186 points) and background points (1029 points).

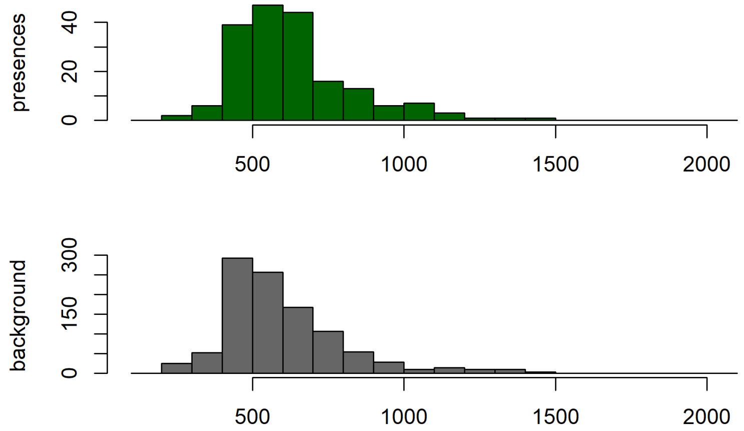

# Supp. File 5 – *Ixodes ricinus* models

This tab provides the list of all models tested for the distribution of *Ixodes ricinus*. The mean and standard deviation (sd) values over the 20 runs are given for each of the evaluation parameters. ***reg*** is the value of the regularization multiplier. ***features*** indicate the features used (l=linear, lp=linear and product, lq = linear and quadratic, lpq=linear product and quadratic). ***OE_test*** is the omission error on the testing dataset and ***OE_indep*** on the independent dataset. **# coeff** is the number of non-zeros coefficients estimated by the model. The ranks (1-4) correspond to the ranking procedure defined in the method section. The final rank gives the final ranking of the models (1=best model, parameters selected for the final modelling).

| Variables Selection | PCA %variance | reg | features | # env. predictors | rank1 AUC_test | rank2 OE_test | rank3 OE_indep | rank4 #coeffs | ranks sum | final rank | mean AUC_test | mean # coeff. | mean OE_test | mean OE_indep | mean AUC_train | mean BIC | sd AUC_test | sd # coeff | sd OE_test | sd OE_indep | sd AUC_train | sd BIC |
|---|---|---|---|---|---|---|---|---|---|---|---|---|---|---|---|---|---|---|---|---|---|---|
| corr/VIF | - | 1 | l | 27 | 48 | 55 | 54 | 33 | 190 | 54 | 0.711 | 23.600 | 0.335 | 0.390 | 0.717 | 29766 | 0.009 | 1.095 | 0.046 | 0.049 | 0.003 | 14.130 |
| corr/VIF | - | 1 | lp | 27 | 41 | 53 | 56 | 55 | 205 | 56 | 0.733 | 92.550 | 0.312 | 0.428 | 0.752 | 30235 | 0.012 | 6.194 | 0.049 | 0.073 | 0.004 | 74.669 |
| corr/VIF | - | 1 | lq | 27 | 38 | 49 | 49 | 40 | 176 | 51 | 0.738 | 32.600 | 0.282 | 0.344 | 0.742 | 29724 | 0.009 | 1.729 | 0.058 | 0.062 | 0.003 | 19.955 |
| corr/VIF | - | 1 | lpq | 27 | 34 | 44 | 53 | 54 | 185 | 53 | 0.747 | 92.200 | 0.270 | 0.377 | 0.768 | 30120 | 0.012 | 5.187 | 0.040 | 0.052 | 0.004 | 53.552 |
| corr/VIF | - | 5 | l | 27 | 49 | 54 | 50 | 13 | 166 | 49 | 0.710 | 14.950 | 0.323 | 0.349 | 0.715 | 29662 | 0.010 | 1.099 | 0.045 | 0.039 | 0.003 | 16.042 |
| corr/VIF | - | 5 | lp | 27 | 46 | 56 | 55 | 37 | 194 | 55 | 0.721 | 28.350 | 0.336 | 0.397 | 0.728 | 29696 | 0.011 | 2.368 | 0.044 | 0.052 | 0.003 | 23.684 |
| corr/VIF | - | 5 | lq | 27 | 42 | 51 | 48 | 27 | 168 | 50 | 0.730 | 20.950 | 0.303 | 0.331 | 0.732 | 29622 | 0.010 | 1.504 | 0.049 | 0.048 | 0.003 | 16.430 |
| corr/VIF | - | 5 | lpq | 27 | 36 | 52 | 52 | 41 | 181 | 52 | 0.741 | 32.700 | 0.305 | 0.360 | 0.746 | 29641 | 0.008 | 2.638 | 0.045 | 0.050 | 0.003 | 25.442 |
| PCA | 50% | 1 | l | 3 | 55 | 2 | 2 | 1 | 60 | 3 | 0.663 | 3.000 | 0.147 | 0.161 | 0.659 | 29756 | 0.010 | 0.000 | 0.049 | 0.042 | 0.004 | 11.505 |
| PCA | 50% | 1 | lp | 3 | 56 | 1 | 1 | 2 | 60 | 4 | 0.660 | 5.600 | 0.140 | 0.154 | 0.660 | 29766 | 0.009 | 0.503 | 0.057 | 0.057 | 0.003 | 14.468 |
| PCA | 50% | 1 | lq | 3 | 52 | 47 | 46 | 3 | 148 | 45 | 0.678 | 5.700 | 0.280 | 0.298 | 0.678 | 29656 | 0.010 | 0.470 | 0.053 | 0.052 | 0.003 | 14.073 |
| PCA | 50% | 1 | lpq | 3 | 53 | 5 | 29 | 5 | 92 | 18 | 0.674 | 7.650 | 0.213 | 0.228 | 0.679 | 29665 | 0.011 | 0.988 | 0.079 | 0.083 | 0.004 | 14.545 |
| PCA | 70% | 1 | l | 6 | 54 | 3 | 15 | 4 | 76 | 8 | 0.666 | 6.000 | 0.185 | 0.211 | 0.667 | 29757 | 0.008 | 0.000 | 0.069 | 0.067 | 0.003 | 9.190 |
| PCA | 70% | 1 | lp | 6 | 51 | 15 | 47 | 21 | 134 | 43 | 0.691 | 19.100 | 0.234 | 0.300 | 0.703 | 29725 | 0.007 | 0.852 | 0.048 | 0.071 | 0.003 | 11.890 |
| PCA | 70% | 1 | lq | 6 | 50 | 13 | 45 | 9 | 117 | 35 | 0.709 | 11.800 | 0.233 | 0.272 | 0.714 | 29591 | 0.011 | 0.410 | 0.068 | 0.086 | 0.004 | 18.314 |
| PCA | 70% | 1 | lpq | 6 | 47 | 32 | 51 | 29 | 159 | 48 | 0.720 | 21.750 | 0.253 | 0.358 | 0.729 | 29588 | 0.008 | 1.209 | 0.065 | 0.107 | 0.003 | 21.109 |
| PCA | 80% | 1 | l | 10 | 44 | 41 | 35 | 8 | 128 | 40 | 0.724 | 10.000 | 0.266 | 0.244 | 0.723 | 29658 | 0.010 | 0.000 | 0.059 | 0.060 | 0.004 | 13.431 |
| PCA | 80% | 1 | lp | 10 | 32 | 36 | 41 | 44 | 153 | 47 | 0.749 | 42.250 | 0.255 | 0.258 | 0.757 | 29766 | 0.010 | 2.403 | 0.042 | 0.052 | 0.003 | 28.257 |
| PCA | 80% | 1 | lq | 10 | 19 | 40 | 38 | 23 | 120 | 38 | 0.763 | 19.600 | 0.265 | 0.251 | 0.766 | 29475 | 0.011 | 0.503 | 0.037 | 0.039 | 0.004 | 25.669 |
| PCA | 80% | 1 | lpq | 10 | 10 | 10 | 27 | 50 | 97 | 22 | 0.780 | 53.000 | 0.230 | 0.224 | 0.793 | 29582 | 0.008 | 1.522 | 0.046 | 0.045 | 0.003 | 23.603 |
| PCA | 80% | 2 | l | 10 | 45 | 42 | 36 | 7 | 130 | 41 | 0.723 | 9.900 | 0.268 | 0.247 | 0.724 | 29644 | 0.010 | 0.308 | 0.060 | 0.049 | 0.003 | 14.195 |
| PCA | 80% | 2 | lp | 10 | 33 | 38 | 39 | 42 | 152 | 46 | 0.747 | 33.450 | 0.262 | 0.254 | 0.754 | 29681 | 0.007 | 1.504 | 0.045 | 0.048 | 0.003 | 15.620 |
| PCA | 80% | 2 | lq | 10 | 23 | 24 | 30 | 22 | 99 | 23 | 0.761 | 19.250 | 0.242 | 0.229 | 0.765 | 29447 | 0.008 | 0.851 | 0.058 | 0.056 | 0.003 | 17.258 |
| PCA | 80% | 2 | lpq | 10 | 11 | 17 | 34 | 46 | 108 | 29 | 0.777 | 44.150 | 0.238 | 0.232 | 0.785 | 29505 | 0.009 | 2.412 | 0.043 | 0.044 | 0.003 | 26.689 |
| PCA | 80% | 5 | l | 10 | 43 | 34 | 31 | 6 | 114 | 33 | 0.724 | 9.400 | 0.254 | 0.229 | 0.722 | 29621 | 0.010 | 0.598 | 0.040 | 0.029 | 0.003 | 15.508 |
| PCA | 80% | 5 | lp | 10 | 39 | 19 | 14 | 19 | 91 | 17 | 0.736 | 17.150 | 0.240 | 0.211 | 0.739 | 29605 | 0.011 | 1.785 | 0.048 | 0.047 | 0.004 | 22.917 |
| PCA | 80% | 5 | lq | 10 | 30 | 18 | 22 | 12 | 82 | 11 | 0.755 | 13.900 | 0.239 | 0.219 | 0.757 | 29410 | 0.011 | 1.210 | 0.050 | 0.047 | 0.004 | 26.847 |
| PCA | 80% | 5 | lpq | 10 | 22 | 27 | 33 | 26 | 108 | 28 | 0.761 | 20.050 | 0.247 | 0.231 | 0.764 | 29410 | 0.012 | 1.276 | 0.057 | 0.058 | 0.004 | 24.385 |
| PCA | 90% | 2 | l | 17 | 31 | 26 | 11 | 18 | 86 | 14 | 0.750 | 17.000 | 0.244 | 0.205 | 0.750 | 29575 | 0.009 | 0.000 | 0.040 | 0.033 | 0.003 | 14.684 |
| PCA | 90% | 2 | lp | 17 | 6 | 9 | 20 | 51 | 86 | 15 | 0.788 | 80.150 | 0.230 | 0.219 | 0.799 | 29805 | 0.010 | 4.171 | 0.041 | 0.034 | 0.004 | 28.721 |
| PCA | 90% | 2 | lq | 17 | 12 | 12 | 37 | 39 | 100 | 25 | 0.776 | 31.400 | 0.232 | 0.251 | 0.785 | 29450 | 0.009 | 0.883 | 0.036 | 0.040 | 0.003 | 18.655 |
| PCA | 90% | 2 | lpq | 17 | 1 | 7 | 24 | 52 | 84 | 12 | 0.804 | 84.500 | 0.221 | 0.223 | 0.820 | 29678 | 0.007 | 2.911 | 0.036 | 0.034 | 0.002 | 33.233 |
| PCA | 90% | 5 | l | 17 | 35 | 22 | 12 | 16 | 85 | 13 | 0.746 | 16.450 | 0.242 | 0.207 | 0.748 | 29544 | 0.010 | 0.759 | 0.032 | 0.028 | 0.004 | 16.702 |
| PCA | 90% | 5 | lp | 17 | 26 | 46 | 32 | 38 | 142 | 44 | 0.758 | 30.950 | 0.274 | 0.230 | 0.771 | 29503 | 0.012 | 2.259 | 0.057 | 0.053 | 0.004 | 31.500 |
| PCA | 90% | 5 | lq | 17 | 17 | 21 | 42 | 31 | 111 | 32 | 0.770 | 22.050 | 0.241 | 0.258 | 0.777 | 29381 | 0.009 | 0.999 | 0.055 | 0.058 | 0.003 | 19.958 |
| PCA | 90% | 5 | lpq | 17 | 8 | 4 | 26 | 43 | 81 | 10 | 0.783 | 34.800 | 0.209 | 0.224 | 0.789 | 29386 | 0.007 | 2.285 | 0.033 | 0.037 | 0.003 | 25.914 |
| PCA | 90% | 10 | l | 17 | 37 | 29 | 21 | 10 | 97 | 20 | 0.739 | 12.200 | 0.252 | 0.219 | 0.740 | 29540 | 0.007 | 0.768 | 0.065 | 0.052 | 0.003 | 14.725 |
| PCA | 90% | 10 | lp | 17 | 40 | 30 | 25 | 15 | 110 | 30 | 0.736 | 15.400 | 0.252 | 0.224 | 0.745 | 29531 | 0.011 | 1.353 | 0.050 | 0.041 | 0.003 | 15.650 |
| PCA | 90% | 10 | lq | 17 | 21 | 33 | 43 | 11 | 108 | 27 | 0.763 | 13.400 | 0.253 | 0.263 | 0.761 | 29396 | 0.008 | 0.883 | 0.042 | 0.038 | 0.003 | 20.097 |
| PCA | 90% | 10 | lpq | 17 | 25 | 37 | 44 | 14 | 120 | 37 | 0.759 | 15.150 | 0.259 | 0.267 | 0.764 | 29391 | 0.012 | 1.496 | 0.051 | 0.044 | 0.004 | 29.106 |
| PCA | 95% | 5 | l | 24 | 20 | 43 | 8 | 30 | 101 | 26 | 0.763 | 21.950 | 0.270 | 0.202 | 0.773 | 29436 | 0.011 | 0.887 | 0.046 | 0.044 | 0.004 | 21.012 |
| PCA | 95% | 5 | lp | 24 | 7 | 31 | 3 | 47 | 88 | 16 | 0.783 | 46.550 | 0.252 | 0.187 | 0.791 | 29492 | 0.011 | 3.605 | 0.041 | 0.036 | 0.004 | 25.573 |
| PCA | 95% | 5 | lq | 24 | 9 | 6 | 5 | 36 | 56 | 1 | 0.781 | 26.750 | 0.220 | 0.195 | 0.789 | 29338 | 0.009 | 1.070 | 0.035 | 0.032 | 0.003 | 23.083 |
| PCA | 95% | 5 | lpq | 24 | 4 | 16 | 9 | 48 | 77 | 9 | 0.795 | 48.700 | 0.235 | 0.203 | 0.806 | 29385 | 0.011 | 3.294 | 0.043 | 0.042 | 0.004 | 33.093 |
| PCA | 95% | 10 | l | 24 | 29 | 48 | 17 | 17 | 111 | 31 | 0.756 | 16.900 | 0.280 | 0.214 | 0.762 | 29445 | 0.009 | 1.165 | 0.031 | 0.023 | 0.003 | 20.928 |
| PCA | 95% | 10 | lp | 24 | 27 | 50 | 28 | 25 | 130 | 42 | 0.758 | 19.800 | 0.286 | 0.225 | 0.764 | 29451 | 0.010 | 1.322 | 0.051 | 0.046 | 0.003 | 15.838 |
| PCA | 95% | 10 | lq | 24 | 16 | 14 | 7 | 20 | 57 | 2 | 0.770 | 18.100 | 0.233 | 0.202 | 0.777 | 29335 | 0.009 | 0.718 | 0.047 | 0.038 | 0.003 | 16.012 |
| PCA | 95% | 10 | lpq | 24 | 18 | 23 | 10 | 24 | 75 | 7 | 0.770 | 19.650 | 0.242 | 0.204 | 0.778 | 29346 | 0.009 | 1.348 | 0.048 | 0.043 | 0.003 | 17.929 |
| PCA | 100% | 5 | l | 69 | 15 | 35 | 4 | 45 | 99 | 24 | 0.773 | 42.550 | 0.255 | 0.187 | 0.784 | 29519 | 0.010 | 2.212 | 0.031 | 0.030 | 0.004 | 27.079 |
| PCA | 100% | 5 | lp | 69 | 3 | 20 | 19 | 53 | 95 | 19 | 0.795 | 91.750 | 0.240 | 0.217 | 0.811 | 29721 | 0.011 | 5.129 | 0.047 | 0.037 | 0.004 | 52.228 |
| PCA | 100% | 5 | lq | 69 | 5 | 8 | 6 | 49 | 68 | 5 | 0.790 | 48.900 | 0.226 | 0.201 | 0.799 | 29432 | 0.008 | 2.654 | 0.033 | 0.045 | 0.003 | 27.838 |
| PCA | 100% | 5 | lpq | 69 | 2 | 25 | 40 | 56 | 123 | 39 | 0.803 | 93.800 | 0.243 | 0.255 | 0.824 | 29622 | 0.008 | 4.895 | 0.029 | 0.036 | 0.003 | 46.856 |
| PCA | 100% | 10 | l | 69 | 24 | 45 | 18 | 28 | 115 | 34 | 0.760 | 21.700 | 0.272 | 0.215 | 0.764 | 29462 | 0.011 | 1.780 | 0.047 | 0.036 | 0.004 | 23.307 |
| PCA | 100% | 10 | lp | 69 | 28 | 39 | 16 | 35 | 118 | 36 | 0.757 | 25.550 | 0.263 | 0.211 | 0.768 | 29467 | 0.010 | 2.911 | 0.036 | 0.030 | 0.003 | 21.902 |
| PCA | 100% | 10 | lq | 69 | 14 | 28 | 23 | 32 | 97 | 21 | 0.774 | 23.150 | 0.248 | 0.222 | 0.777 | 29375 | 0.009 | 1.725 | 0.040 | 0.030 | 0.003 | 21.992 |
| PCA | 100% | 10 | lpq | 69 | 13 | 11 | 13 | 34 | 71 | 6 | 0.774 | 25.450 | 0.230 | 0.210 | 0.779 | 29382 | 0.008 | 1.959 | 0.030 | 0.030 | 0.004 | 26.113 |

## Supp. File 6 – *Ixodes ricinus* suitability maps

Maps of suitability predicted based on the “best” model presented in the paper.

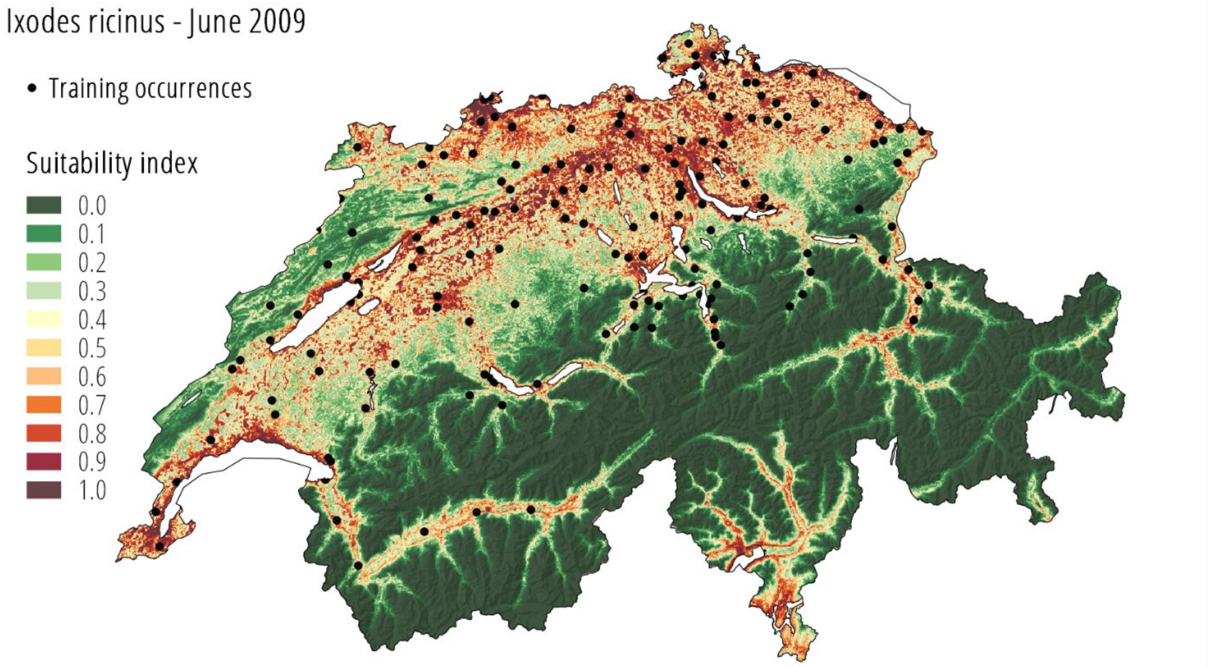


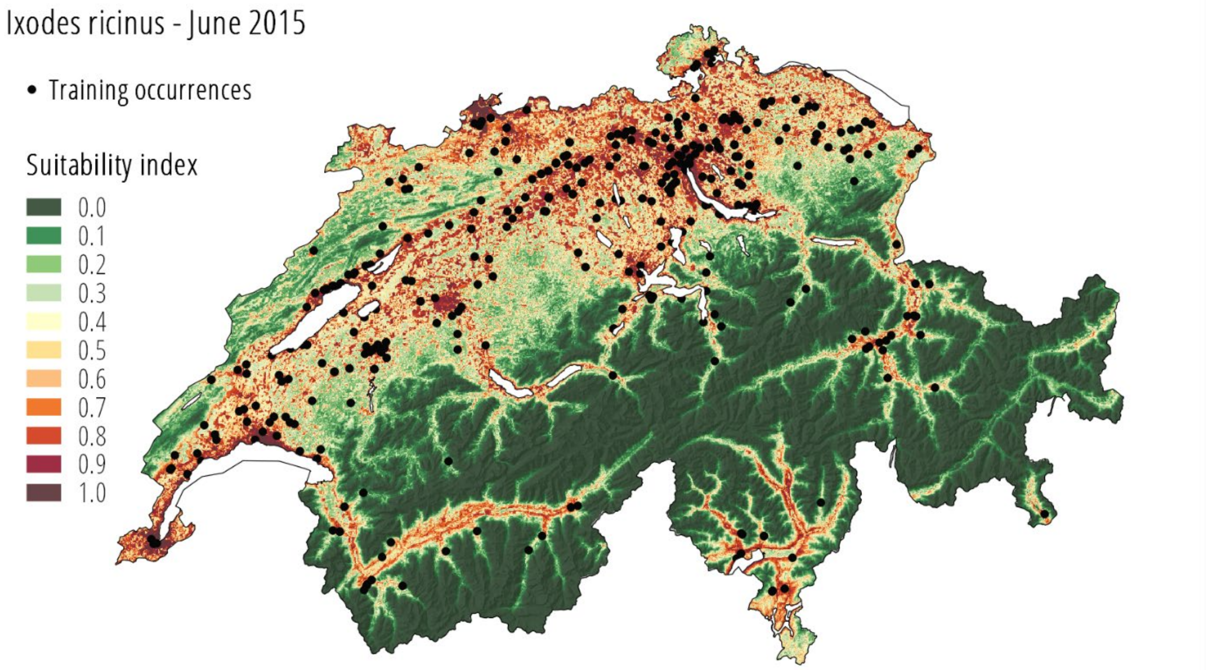

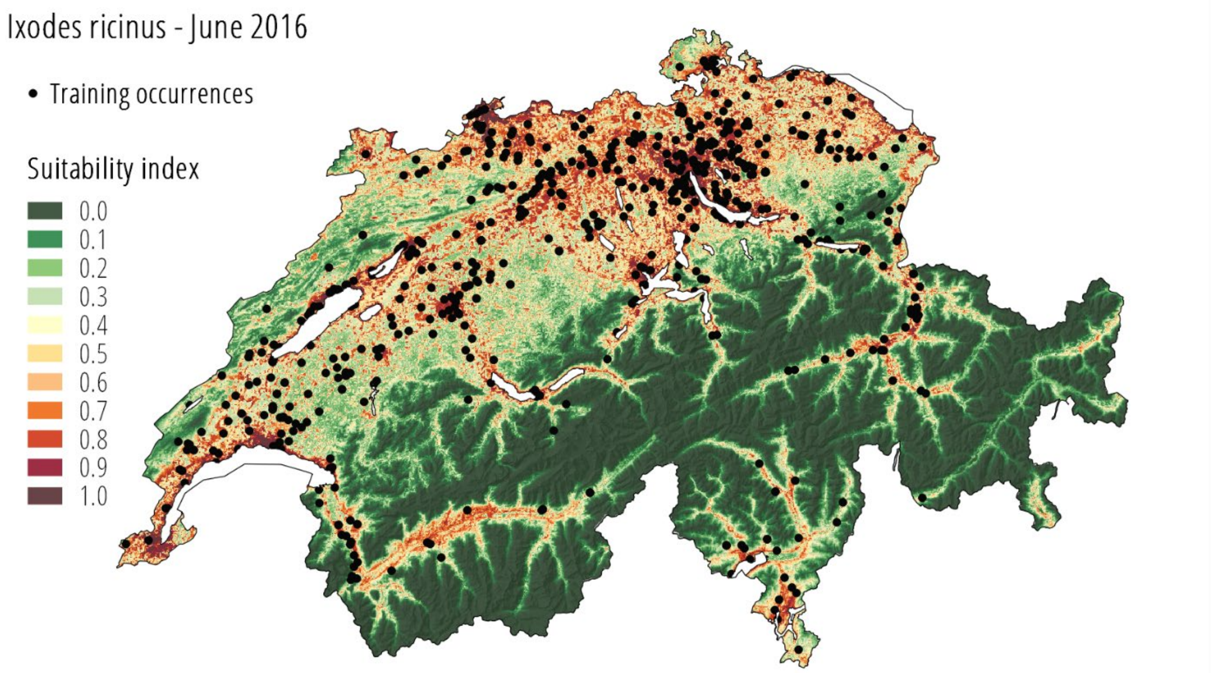
Ixodes ricinus - June 2016
Training occurrences
Suitability index
0.0
0.1
0.2
0.3
0.4
0.5
0.6
0.7
0.8
0.9
1.0

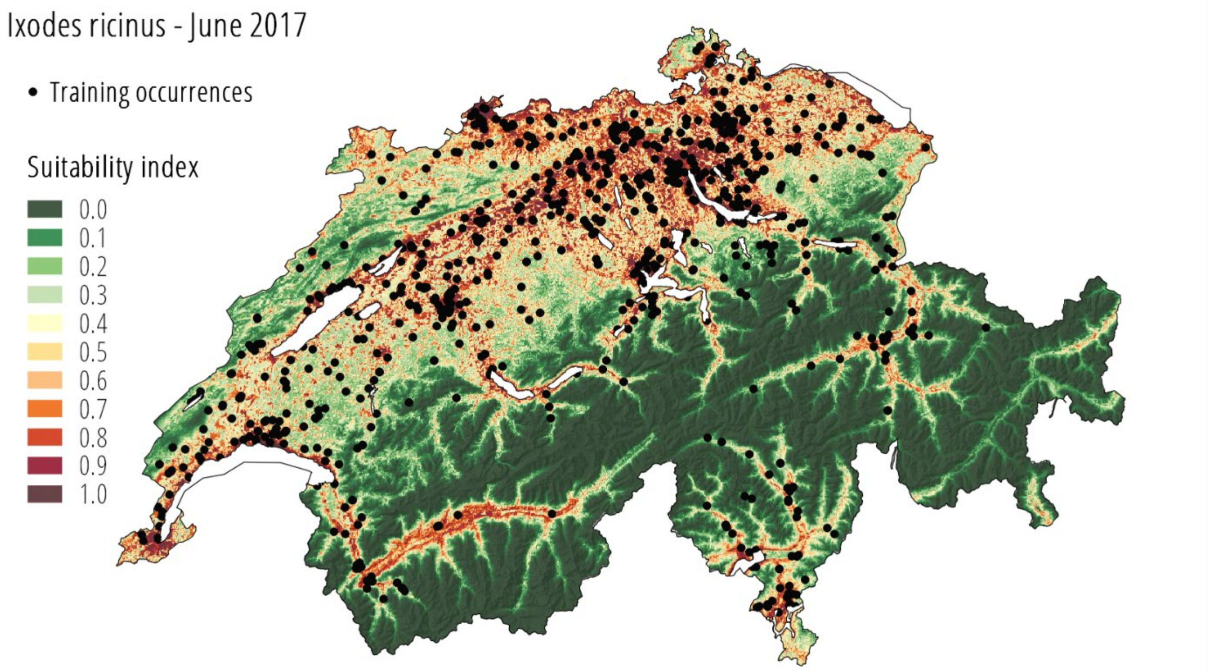
Ixodes ricinus - June 2017
Training occurrences
Suitability index
0.0
0.1
0.2
0.3
0.4
0.5
0.6
0.7
0.8
0.9
1.0

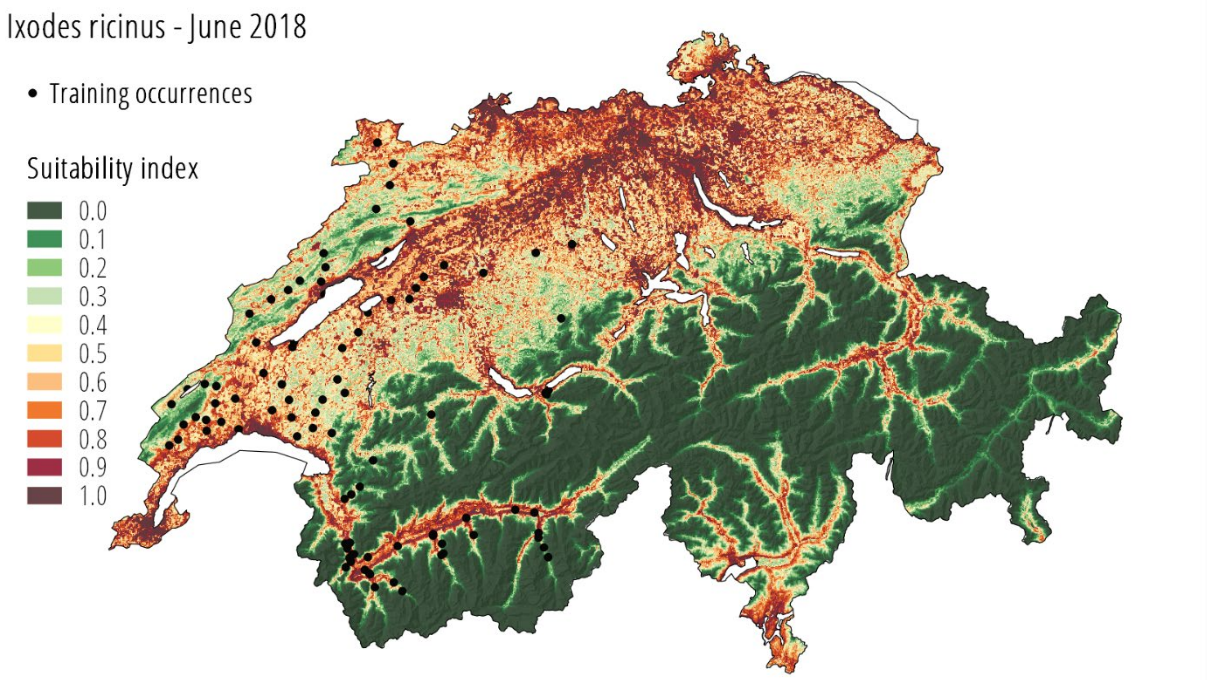
Ixodes ricinus - June 2018
• Training occurrences
Suitability index
0.0
0.1
0.2
0.3
0.4
0.5
0.6
0.7
0.8
0.9
1.0

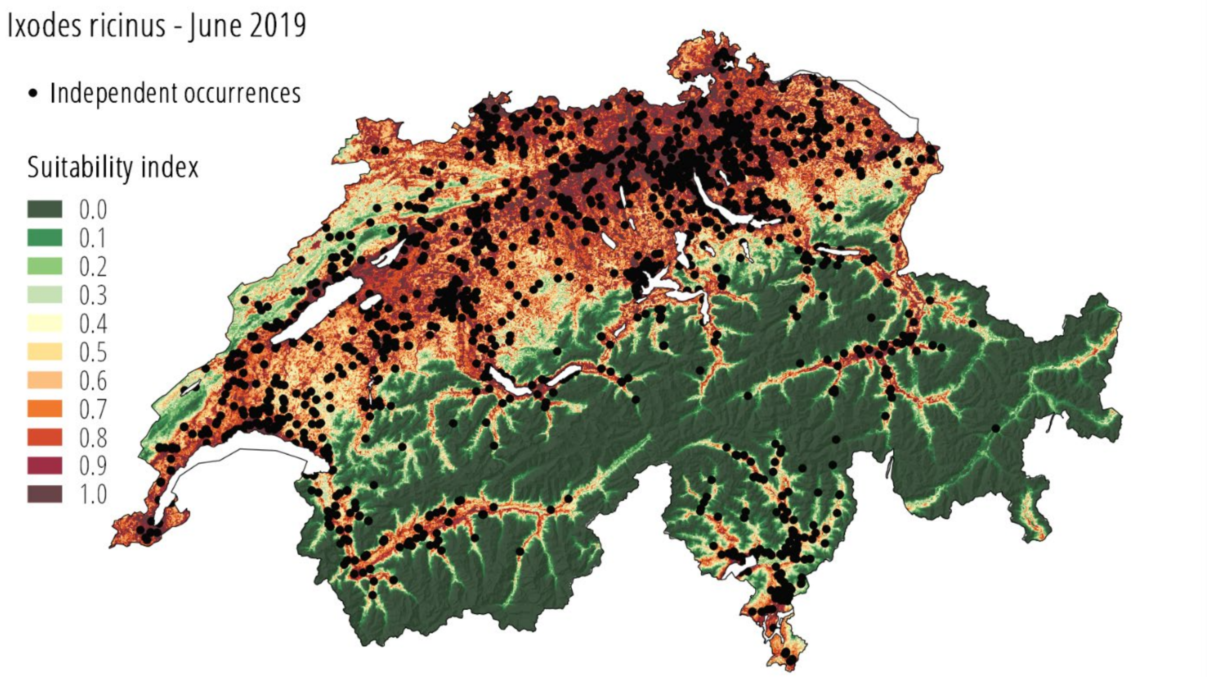
Ixodes ricinus - June 2019
• Independent occurrences
Suitability index
0.0
0.1
0.2
0.3
0.4
0.5
0.6
0.7
0.8
0.9
1.0

# Supp. File 7 – *Chlamydiales* models

This tab provides the list of all models tested for the distribution of *Chlamydiales*. The mean and standard deviation (sd) values over the 20 runs are given for each of the evaluation parameters. ***reg***. Is the value of the regularization parameter. ***feat.*** indicates the features used (l=linear, lp=linear and product, lq = linear and quadratic, lpq=linear product and quadratic). ***med suit. P 2009*** (resp. 2018) is the median of the suitability predicted on presences points from 2009 (resp. 2018). **med suit. "A" 2009** (resp. 2018) is the median of the suitability predicted at sites where no *Chlamydiales* were found ("absences") in 2009 (resp. 2018). **# coeff** is the number of non-zeros coefficients estimated by the model. The ranks (1-4) correspond to the ranking procedure defined in the method section. The final rank givse the final ranking of the models (1=best model, parameters selected for the final modelling).

| Variables Selection | PCA %var. | reg | feat. | # env | rank1 AUCtest | rank2 diff 2009 | rank3 diff 2018 | rank4 #coeffs | sum of ranks | final rank | mean AUCtest | mean #coeff. | mean med suit. P 2009 | mean med suit. "A" 2009 | diff 2009 | mean med suit. P 2018 | mean med suit."A"2018 | diff 2018 | mean AUCtrain | mean BIC |
|---|---|---|---|---|---|---|---|---|---|---|---|---|---|---|---|---|---|---|---|---|
| corr/VIF | - | 1 | l | 26 | 15 | 32 | 32 | 26 | 105 | 24 | 0.699 | 23.65 | 0.407 | 0.322 | 0.085 | 0.429 | 0.234 | 0.195 | 0.733 | 1958.07 |
| corr/VIF | - | 1 | lp | 26 | 21 | 4 | 6 | 53 | 84 | 6 | 0.693 | 118.20 | 0.477 | 0.241 | 0.235 | 0.470 | 0.073 | 0.397 | 0.846 | 3152.50 |
| corr/VIF | - | 1 | lq | 26 | 1 | 16 | 15 | 35 | 67 | 1 | 0.744 | 36.20 | 0.467 | 0.346 | 0.121 | 0.458 | 0.150 | 0.308 | 0.789 | 2200.49 |
| corr/VIF | - | 1 | lpq | 26 | 23 | 3 | 4 | 54 | 84 | 7 | 0.690 | 121.55 | 0.485 | 0.242 | 0.243 | 0.479 | 0.058 | 0.421 | 0.863 | 3128.88 |
| corr/VIF | - | 5 | l | 26 | 32 | 31 | 34 | 20 | 117 | 34 | 0.679 | 17.90 | 0.422 | 0.336 | 0.085 | 0.450 | 0.263 | 0.186 | 0.732 | 1923.77 |
| corr/VIF | - | 5 | lp | 26 | 5 | 23 | 28 | 40 | 96 | 15 | 0.714 | 41.70 | 0.445 | 0.341 | 0.104 | 0.416 | 0.195 | 0.221 | 0.786 | 2044.63 |
| corr/VIF | - | 5 | lq | 26 | 2 | 33 | 27 | 25 | 87 | 10 | 0.730 | 22.60 | 0.460 | 0.376 | 0.084 | 0.470 | 0.246 | 0.223 | 0.771 | 1920.40 |
| corr/VIF | - | 5 | lpq | 26 | 3 | 24 | 24 | 42 | 93 | 13 | 0.726 | 46.35 | 0.462 | 0.359 | 0.103 | 0.440 | 0.192 | 0.248 | 0.794 | 2099.72 |
| PCA | 50% | 1 | l | 4 | 56 | 52 | 55 | 1 | 164 | 55 | 0.584 | 3.00 | 0.407 | 0.358 | 0.049 | 0.490 | 0.424 | 0.066 | 0.604 | 1902.96 |
| PCA | 50% | 1 | lp | 4 | 55 | 51 | 56 | 3 | 165 | 56 | 0.595 | 5.75 | 0.413 | 0.363 | 0.050 | 0.467 | 0.416 | 0.051 | 0.602 | 1916.03 |
| PCA | 50% | 1 | lq | 4 | 54 | 47 | 49 | 4 | 154 | 52 | 0.611 | 5.95 | 0.417 | 0.361 | 0.056 | 0.453 | 0.314 | 0.139 | 0.653 | 1902.24 |
| PCA | 50% | 1 | lpq | 4 | 52 | 35 | 53 | 7 | 147 | 50 | 0.622 | 8.55 | 0.426 | 0.343 | 0.083 | 0.422 | 0.293 | 0.129 | 0.650 | 1913.39 |
| PCA | 70% | 1 | l | 6 | 53 | 42 | 54 | 2 | 151 | 51 | 0.622 | 4.95 | 0.414 | 0.348 | 0.066 | 0.490 | 0.369 | 0.121 | 0.640 | 1901.84 |
| PCA | 70% | 1 | lp | 6 | 51 | 45 | 52 | 15 | 163 | 54 | 0.637 | 14.15 | 0.408 | 0.348 | 0.060 | 0.474 | 0.342 | 0.132 | 0.654 | 1949.90 |
| PCA | 70% | 1 | lq | 6 | 39 | 41 | 42 | 9 | 131 | 39 | 0.673 | 9.70 | 0.438 | 0.369 | 0.069 | 0.477 | 0.307 | 0.170 | 0.714 | 1897.24 |
| PCA | 70% | 1 | lpq | 6 | 35 | 40 | 41 | 21 | 137 | 45 | 0.676 | 18.20 | 0.443 | 0.371 | 0.072 | 0.450 | 0.280 | 0.170 | 0.719 | 1941.72 |
| PCA | 80% | 1 | l | 9 | 49 | 34 | 45 | 6 | 134 | 41 | 0.652 | 7.90 | 0.417 | 0.334 | 0.083 | 0.468 | 0.317 | 0.151 | 0.672 | 1906.33 |
| PCA | 80% | 1 | lp | 9 | 16 | 27 | 25 | 33 | 101 | 19 | 0.697 | 33.15 | 0.393 | 0.299 | 0.093 | 0.448 | 0.205 | 0.244 | 0.756 | 2144.63 |
| PCA | 80% | 1 | lq | 9 | 25 | 26 | 31 | 16 | 98 | 16 | 0.687 | 15.30 | 0.416 | 0.318 | 0.098 | 0.446 | 0.250 | 0.196 | 0.729 | 2013.86 |
| PCA | 80% | 1 | lpq | 9 | 6 | 30 | 16 | 37 | 89 | 11 | 0.708 | 39.10 | 0.401 | 0.314 | 0.087 | 0.450 | 0.148 | 0.301 | 0.781 | 2303.41 |
| PCA | 90% | 1 | l | 14 | 46 | 38 | 37 | 13 | 134 | 42 | 0.665 | 12.60 | 0.412 | 0.333 | 0.079 | 0.452 | 0.273 | 0.179 | 0.694 | 1921.21 |
| PCA | 90% | 1 | lp | 14 | 47 | 6 | 17 | 49 | 119 | 35 | 0.663 | 79.50 | 0.437 | 0.235 | 0.202 | 0.393 | 0.108 | 0.285 | 0.790 | 3111.37 |
| PCA | 90% | 1 | lq | 14 | 27 | 46 | 33 | 29 | 135 | 43 | 0.685 | 24.85 | 0.404 | 0.346 | 0.058 | 0.410 | 0.223 | 0.187 | 0.748 | 2052.67 |
| PCA | 90% | 1 | lpq | 14 | 12 | 5 | 8 | 50 | 75 | 2 | 0.702 | 87.20 | 0.441 | 0.232 | 0.209 | 0.417 | 0.080 | 0.337 | 0.815 | 3053.53 |
| PCA | 90% | 2 | l | 14 | 38 | 39 | 39 | 12 | 128 | 37 | 0.674 | 12.25 | 0.418 | 0.344 | 0.074 | 0.455 | 0.284 | 0.172 | 0.692 | 1916.91 |
| PCA | 90% | 2 | lp | 14 | 24 | 10 | 19 | 45 | 98 | 17 | 0.688 | 67.65 | 0.441 | 0.268 | 0.173 | 0.431 | 0.150 | 0.281 | 0.780 | 2956.55 |
| PCA | 90% | 2 | lq | 14 | 11 | 48 | 38 | 28 | 125 | 36 | 0.703 | 24.15 | 0.427 | 0.371 | 0.055 | 0.420 | 0.245 | 0.175 | 0.741 | 1977.26 |
| PCA | 90% | 2 | lpq | 14 | 34 | 9 | 14 | 47 | 104 | 22 | 0.676 | 76.10 | 0.454 | 0.274 | 0.180 | 0.431 | 0.121 | 0.310 | 0.816 | 2985.31 |
| PCA | 90% | 5 | l | 14 | 43 | 37 | 44 | 11 | 135 | 44 | 0.668 | 11.55 | 0.435 | 0.356 | 0.080 | 0.463 | 0.306 | 0.157 | 0.686 | 1912.04 |
| PCA | 90% | 5 | lp | 14 | 17 | 14 | 23 | 41 | 95 | 14 | 0.697 | 42.90 | 0.469 | 0.327 | 0.141 | 0.467 | 0.213 | 0.253 | 0.762 | 2245.18 |
| PCA | 90% | 5 | lq | 14 | 26 | 49 | 43 | 23 | 141 | 49 | 0.686 | 21.15 | 0.439 | 0.387 | 0.052 | 0.440 | 0.275 | 0.165 | 0.744 | 1949.26 |
| PCA | 90% | 5 | lpq | 14 | 4 | 13 | 21 | 43 | 81 | 4 | 0.715 | 49.35 | 0.493 | 0.352 | 0.142 | 0.465 | 0.207 | 0.257 | 0.791 | 2266.37 |
| PCA | 90% | 10 | l | 14 | 48 | 36 | 48 | 8 | 140 | 48 | 0.663 | 9.45 | 0.448 | 0.368 | 0.081 | 0.466 | 0.324 | 0.141 | 0.676 | 1901.83 |
| PCA | 90% | 10 | lp | 14 | 33 | 17 | 35 | 27 | 112 | 32 | 0.678 | 23.95 | 0.477 | 0.359 | 0.118 | 0.470 | 0.285 | 0.185 | 0.728 | 1990.70 |
| PCA | 90% | 10 | lq | 14 | 30 | 44 | 47 | 18 | 139 | 46 | 0.681 | 15.65 | 0.462 | 0.401 | 0.061 | 0.456 | 0.308 | 0.148 | 0.724 | 1910.89 |
| PCA | 90% | 10 | lpq | 14 | 19 | 20 | 36 | 30 | 105 | 25 | 0.694 | 26.75 | 0.481 | 0.374 | 0.107 | 0.474 | 0.292 | 0.181 | 0.753 | 2000.00 |
| PCA | 95% | 5 | l | 19 | 36 | 29 | 46 | 17 | 128 | 38 | 0.675 | 15.35 | 0.435 | 0.348 | 0.088 | 0.425 | 0.277 | 0.149 | 0.700 | 1924.01 |
| PCA | 95% | 5 | lp | 19 | 41 | 11 | 18 | 46 | 116 | 33 | 0.671 | 73.50 | 0.489 | 0.319 | 0.170 | 0.467 | 0.186 | 0.281 | 0.802 | 2591.90 |
| PCA | 95% | 5 | lq | 19 | 18 | 50 | 40 | 31 | 139 | 47 | 0.695 | 29.35 | 0.452 | 0.401 | 0.051 | 0.423 | 0.253 | 0.171 | 0.747 | 2034.82 |
| PCA | 95% | 5 | lpq | 19 | 28 | 12 | 11 | 48 | 99 | 18 | 0.683 | 78.90 | 0.496 | 0.354 | 0.142 | 0.498 | 0.174 | 0.324 | 0.827 | 2717.13 |
| PCA | 95% | 10 | l | 19 | 40 | 28 | 50 | 14 | 132 | 40 | 0.672 | 12.90 | 0.453 | 0.366 | 0.088 | 0.452 | 0.316 | 0.137 | 0.689 | 1911.28 |
| PCA | 95% | 10 | lp | 19 | 29 | 15 | 29 | 34 | 107 | 27 | 0.682 | 35.95 | 0.486 | 0.363 | 0.123 | 0.479 | 0.277 | 0.202 | 0.744 | 2058.26 |
| PCA | 95% | 10 | lq | 19 | 37 | 43 | 51 | 24 | 155 | 53 | 0.675 | 21.20 | 0.471 | 0.406 | 0.065 | 0.430 | 0.296 | 0.134 | 0.735 | 1938.55 |
| PCA | 95% | 10 | lpq | 19 | 13 | 25 | 30 | 39 | 107 | 28 | 0.700 | 40.05 | 0.496 | 0.394 | 0.103 | 0.476 | 0.279 | 0.197 | 0.768 | 2145.56 |
| PCA | 100% | 5 | l | 47 | 8 | 19 | 20 | 36 | 83 | 5 | 0.704 | 37.90 | 0.435 | 0.323 | 0.112 | 0.458 | 0.177 | 0.281 | 0.771 | 2063.43 |
| PCA | 100% | 5 | lp | 47 | 50 | 1 | 2 | 55 | 108 | 29 | 0.639 | 211.95 | 0.674 | 0.292 | 0.382 | 0.583 | 0.098 | 0.485 | 0.918 | 3663.34 |
| PCA | 100% | 5 | lq | 47 | 7 | 21 | 13 | 44 | 85 | 8 | 0.708 | 59.40 | 0.444 | 0.337 | 0.107 | 0.481 | 0.166 | 0.315 | 0.798 | 2214.48 |
| PCA | 100% | 5 | lpq | 47 | 44 | 2 | 1 | 56 | 103 | 21 | 0.668 | 219.40 | 0.680 | 0.310 | 0.370 | 0.582 | 0.096 | 0.486 | 0.922 | 3676.00 |
| PCA | 100% | 10 | l | 47 | 9 | 18 | 26 | 32 | 85 | 9 | 0.704 | 30.55 | 0.473 | 0.356 | 0.116 | 0.482 | 0.247 | 0.235 | 0.754 | 1975.76 |
| PCA | 100% | 10 | lp | 47 | 42 | 7 | 9 | 52 | 110 | 30 | 0.668 | 113.05 | 0.537 | 0.342 | 0.196 | 0.532 | 0.196 | 0.336 | 0.850 | 2576.08 |
| PCA | 100% | 10 | lq | 47 | 10 | 22 | 22 | 38 | 92 | 12 | 0.703 | 39.80 | 0.483 | 0.378 | 0.105 | 0.496 | 0.240 | 0.256 | 0.778 | 2020.73 |
| PCA | 100% | 10 | lpq | 47 | 31 | 8 | 12 | 51 | 102 | 20 | 0.680 | 110.85 | 0.548 | 0.364 | 0.184 | 0.531 | 0.209 | 0.322 | 0.855 | 2628.10 |

## Supp. File 8 - *Chlamydiales* : T-test and selection of variables

For the signification of the acronym names, please refer to Supp. File A2.3.

### T-test

For each variable and buffer radius, the heatmap below shows the results of the T-test. Only results that were significant according to the p-value of the T-test are shown (grey area = no significant results). The numbers on the cells indicate the time period considered before sampling date (in number of months) which resulted in the highest T-value for the given combination of variable and buffer radius. Numerical values are available in the following table.

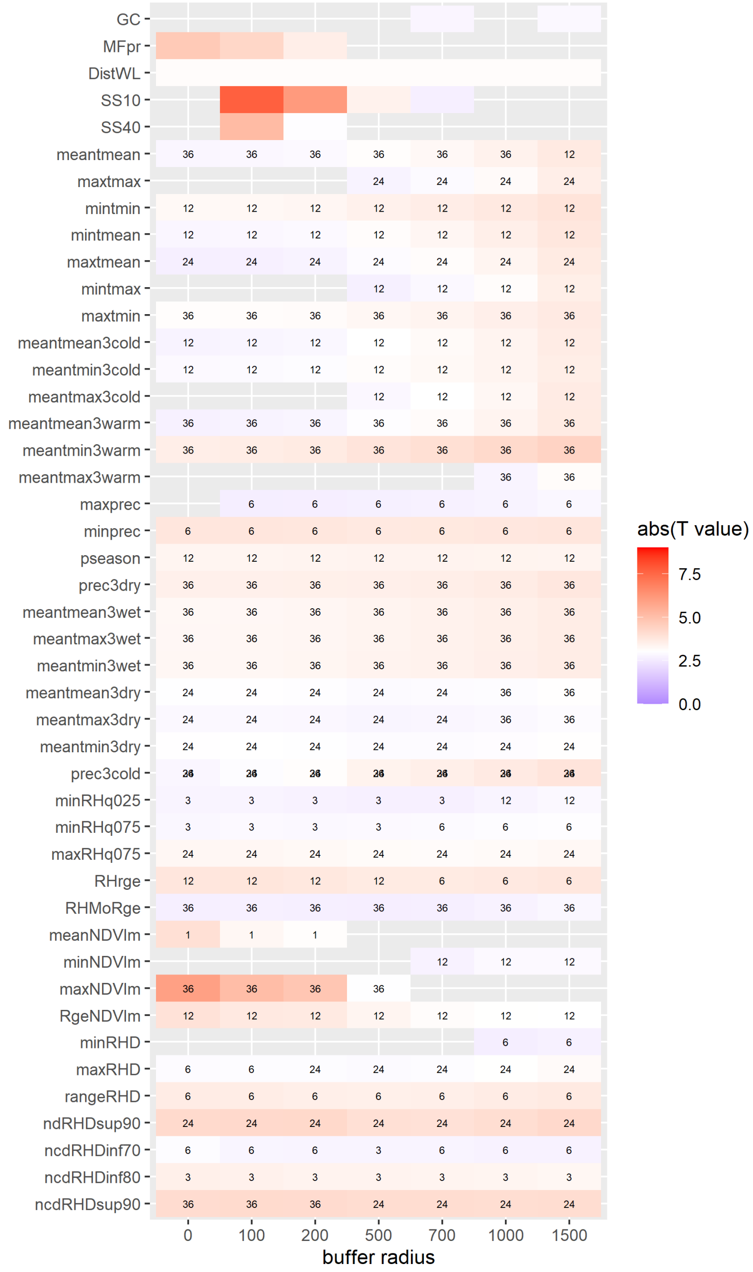

| variable | buffer | time period (months) | mean1 | sd1 | mean0 | sd0 | P-value | T-value |
|---|---|---|---|---|---|---|---|---|
| SS10 | B100m | | 7.76 | 11.79 | 16.76 | 25.282 | 6.49E-14 | 7.70 |
| maxNDVIm | P | 36 | 8335.76 | 530.03 | 8052.00 | 883.681 | 5.65E-09 | -5.96 |
| SS40 | B100m | | 62.72 | 21.58 | 53.03 | 32.526 | 4.12E-07 | -5.16 |
| MFpr | P | | 39.37 | 30.52 | 27.86 | 31.863 | 4.21E-06 | -4.70 |
| meantmin3warm | B1500m | 36 | 12.87 | 0.95 | 13.21 | 1.040 | 1.52E-05 | 4.40 |
| ndRHDsup90 | B200m | 24 | 21.11 | 12.51 | 25.24 | 10.579 | 3.29E-05 | 4.23 |
| ncdRHDsup90 | B100m | 36 | 3.12 | 1.35 | 3.57 | 1.328 | 4.44E-05 | 4.16 |
| meanNDVIm | P | 1 | 7296.71 | 940.89 | 6985.76 | 1156.039 | 8.18E-05 | -3.99 |
| RgeNDVIm | P | 12 | 6378.89 | 1537.42 | 5883.24 | 1788.431 | 1.02E-04 | -3.94 |
| mintmin | B1500m | 12 | -4.82 | 1.25 | -4.43 | 1.277 | 1.31E-04 | 3.88 |
| prec3cold | B1500m | 24 | 24.16 | 11.73 | 20.70 | 7.944 | 1.44E-04 | -3.87 |
| prec3cold | B1500m | 36 | 24.16 | 11.73 | 20.70 | 7.944 | 1.44E-04 | -3.87 |
| RHrge | B100m | 12 | 31.09 | 4.69 | 32.52 | 4.758 | 1.62E-04 | 3.83 |
| minprec | B1500m | 6 | 2.80 | 0.97 | 2.50 | 1.022 | 1.62E-04 | -3.83 |
| mintmean | B1500m | 12 | -2.23 | 1.07 | -1.90 | 1.114 | 1.87E-04 | 3.79 |
| prec3dry | B1500m | 36 | 13.99 | 3.25 | 13.00 | 3.443 | 2.00E-04 | -3.77 |
| meantmean | B1500m | 12 | 9.14 | 1.13 | 9.47 | 1.200 | 2.60E-04 | 3.70 |
| rangeRHD | B1500m | 6 | 44.68 | 4.29 | 45.95 | 4.292 | 2.62E-04 | 3.70 |
| maxtmin | B1500m | 36 | 15.26 | 1.19 | 15.61 | 1.083 | 2.81E-04 | 3.69 |
| maxtmean | B1500m | 24 | 18.01 | 1.21 | 18.37 | 1.300 | 2.90E-04 | 3.67 |
| meantmean3warm | B1500m | 36 | 18.08 | 1.19 | 18.43 | 1.293 | 2.90E-04 | 3.67 |
| meantmax3cold | B1500m | 12 | 2.57 | 1.04 | 2.89 | 1.264 | 2.97E-04 | 3.66 |
| meantmean3cold | B1500m | 12 | -0.36 | 1.09 | -0.03 | 1.259 | 3.48E-04 | 3.62 |
| meantmax3wet | B1500m | 36 | 18.00 | 6.71 | 19.84 | 4.413 | 3.78E-04 | 3.61 |
| meantmin3wet | B1500m | 36 | 9.03 | 5.41 | 10.51 | 3.595 | 3.84E-04 | 3.61 |
| meantmean3wet | B1500m | 36 | 13.29 | 5.98 | 14.93 | 3.945 | 3.85E-04 | 3.61 |
| meantmin3cold | B1500m | 12 | -3.12 | 1.20 | -2.77 | 1.354 | 4.18E-04 | 3.57 |
| maxtmax | B1500m | 24 | 23.61 | 1.45 | 24.02 | 1.518 | 4.49E-04 | 3.55 |
| mintmax | B1500m | 12 | 0.59 | 1.00 | 0.88 | 1.133 | 4.54E-04 | 3.55 |
| ncdRHDinf80 | P | 3 | 29.71 | 9.04 | 27.14 | 9.929 | 5.31E-04 | -3.51 |
| pseason | B500m | 12 | 47.02 | 10.59 | 44.24 | 8.316 | 8.06E-04 | -3.40 |
| maxRHq075 | P | 24 | 87.44 | 3.05 | 88.22 | 2.569 | 1.15E-03 | 3.29 |
| maxRHD | B1500m | 24 | 94.83 | 1.67 | 95.24 | 1.330 | 1.68E-03 | 3.18 |
| meantmax3warm | B1500m | 36 | 23.81 | 1.42 | 24.16 | 1.498 | 1.89E-03 | 3.14 |
| DistWL | B1000m | | 2554.14 | 2311.95 | 3163.80 | 3062.125 | 1.89E-03 | 3.13 |
| meantmean3dry | B1500m | 36 | 4.37 | 3.72 | 5.27 | 3.591 | 2.44E-03 | 3.06 |
| meantmin3dry | B1500m | 24 | 7.54 | 4.17 | 8.55 | 4.132 | 2.53E-03 | 3.05 |
| minRHq075 | B1500m | 6 | 67.80 | 2.81 | 67.12 | 3.235 | 3.19E-03 | -2.97 |
| ncdRHDinf70 | P | 6 | 16.00 | 6.65 | 14.44 | 6.435 | 3.37E-03 | -2.96 |
| meantmax3dry | B1500m | 36 | 0.90 | 3.47 | 1.71 | 3.268 | 3.59E-03 | 2.94 |
| minNDVIm | B1000m | 12 | 1562.21 | 1297.28 | 1866.53 | 1461.392 | 4.22E-03 | 2.89 |
| minRHq025 | B1500m | 12 | 56.33 | 3.66 | 55.49 | 3.983 | 4.66E-03 | -2.85 |
| GC | B1500m | | 0.00 | 0.00 | 0.00 | 0.000 | 4.76E-03 | 2.85 |
| RHMoRge | B1500m | 36 | 11.81 | 1.62 | 12.17 | 1.544 | 5.11E-03 | 2.83 |
| maxprec | B1500m | 6 | 16.33 | 6.61 | 14.89 | 4.944 | 5.19E-03 | -2.82 |
| minRHD | B1500m | 6 | 49.55 | 3.37 | 48.82 | 3.792 | 7.75E-03 | -2.68 |

***mean1*** is the mean of the values for occurrences points, ***mean0*** the mean of the values for background points, ***sd1*** the standard deviation of the values for occurrences points and ***sd0*** the standard deviation of the values for background points.

### Uncorrelated variables used in the model

1. MFpr_P
2. ncdRHDinf80_P_3
3. ncdRHDinf70_P_6
4. maxRHq075_P_24
5. SS10_B100m
6. SS40_B100m
7. ncdRHDsup90_B100m_36
8. ndRHDsup90_B200m_24
9. DistWL_B1000m
10. GC_B1500m
11. minRHq075_B1500m_6
12. rangeRHD_B1500m_6
13. prec3cold_B1500m_24
14. maxRHD_B1500m_24
15. prec3dry_B1500m_36
16. meantmax3wet_B1500m_36
17. meantmean3dry_B1500m_36

## Supp. File 9 - Infection rates

### Infection rate prospective campaign: spatial distribution

The infection rate indicates no spatial clustering.

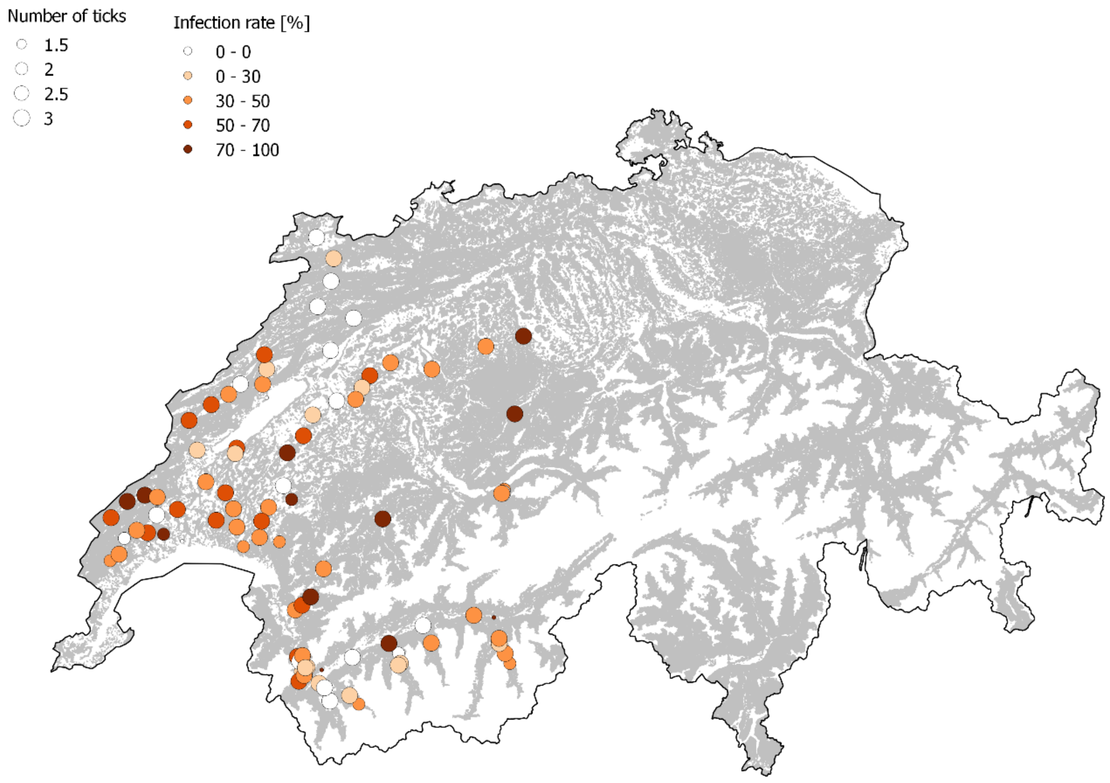

**CT value as a function of sampling date**

Results indicate no concentration of positive values for a given sampling date or a succession of dates. Negative results are also obtained for each sampling date.

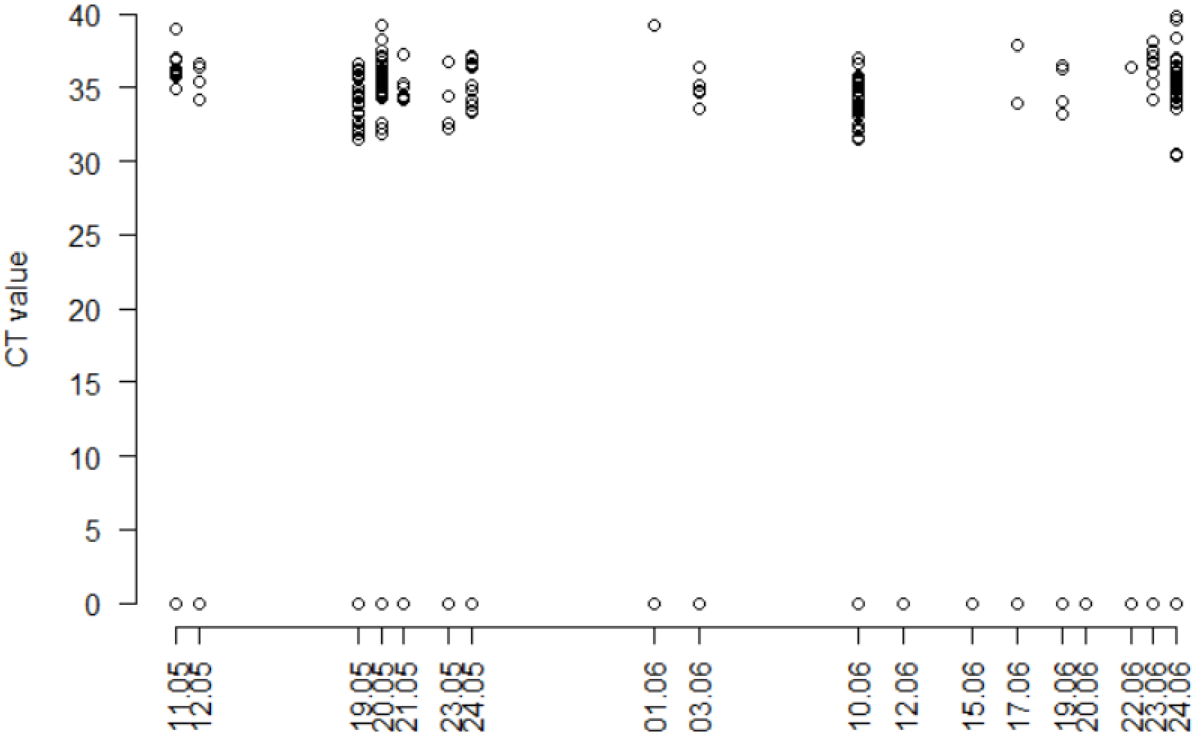


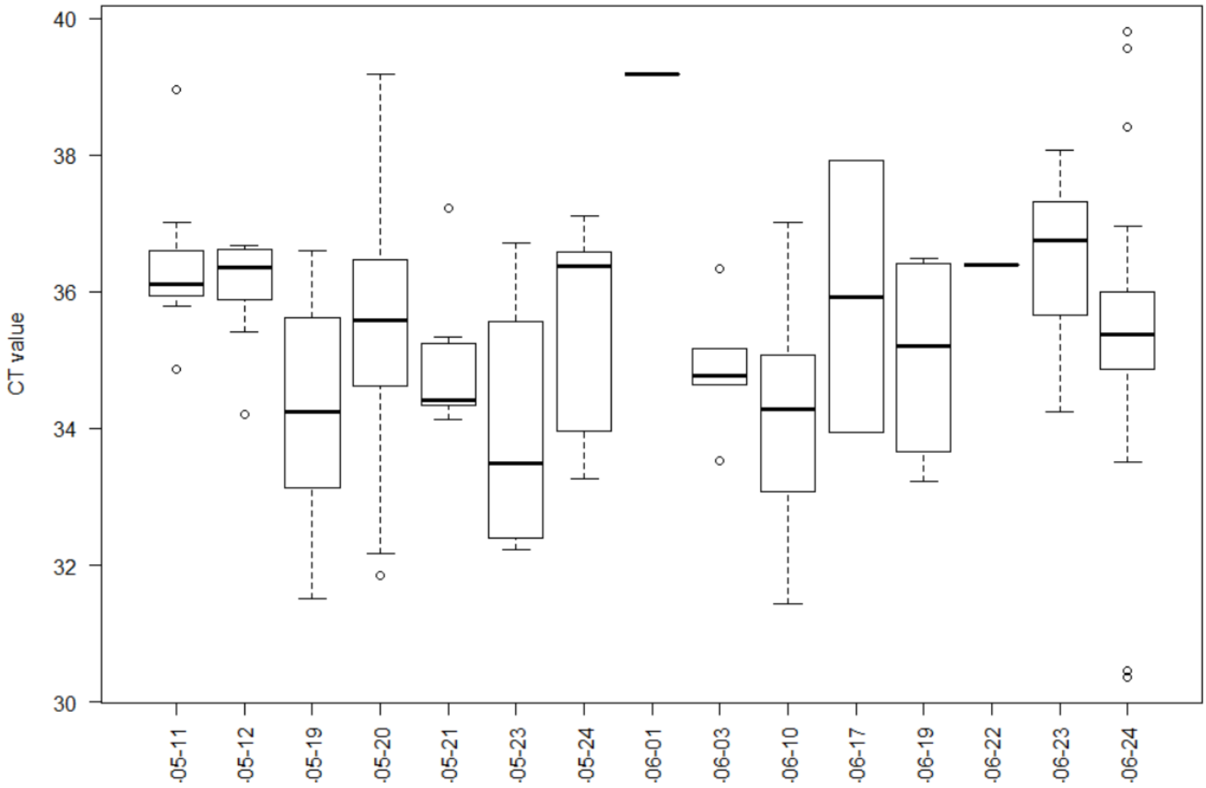

**CT value vs Plate**

Results indicate no concentration of positive values for some plates. Negative results are also obtained on each plate.

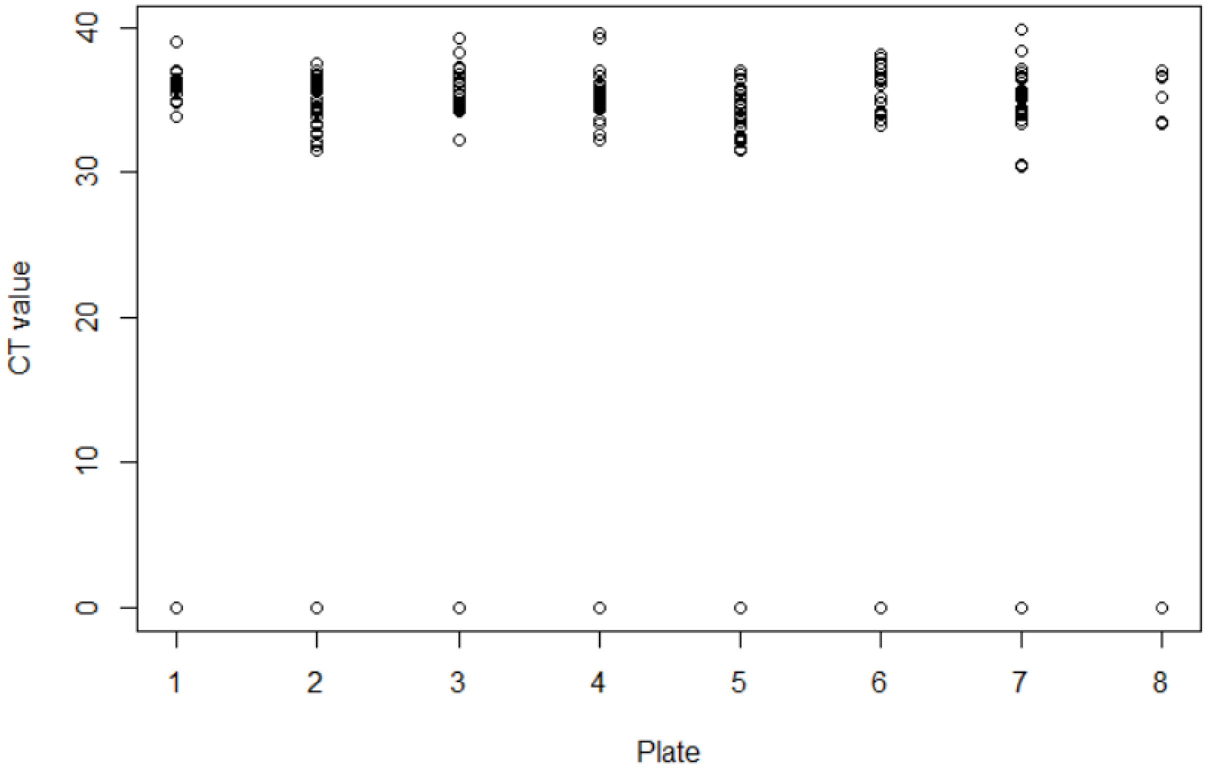


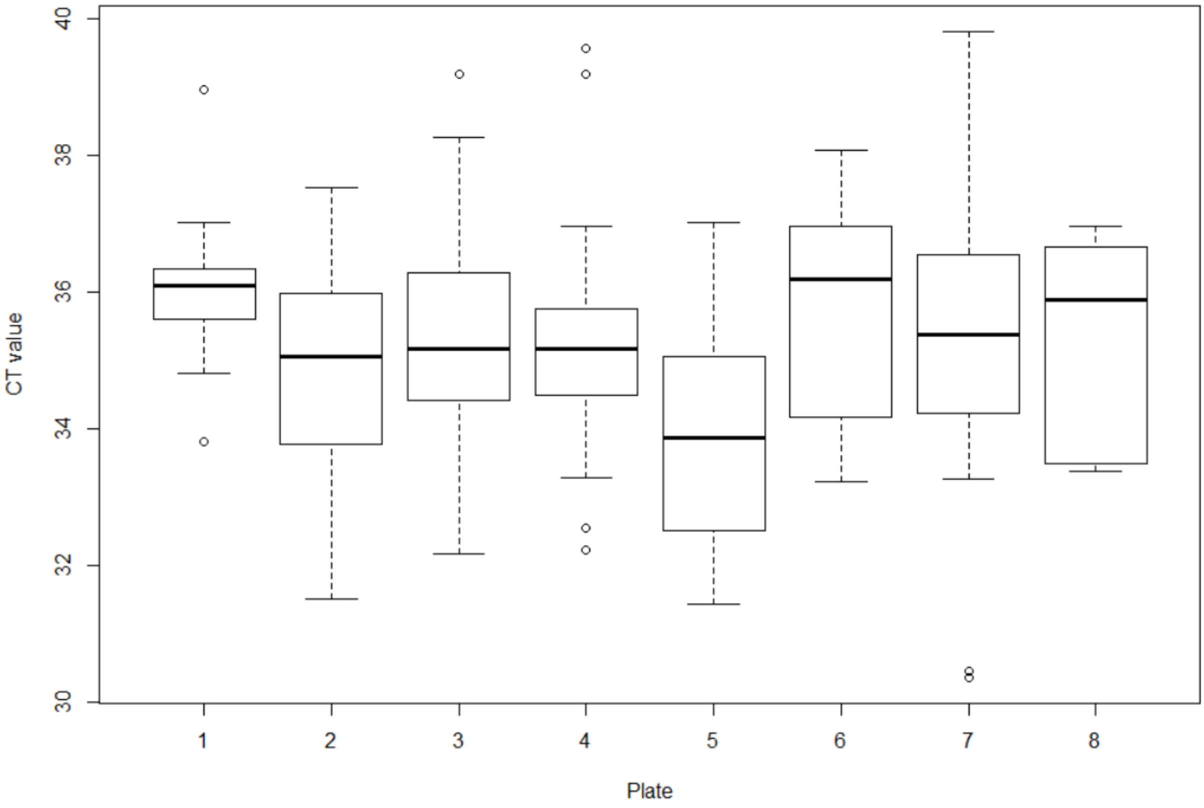

**CT value vs DNA Extraction Date**

Results indicate no concentration of positive values for a given DNA-extraction date. Negative results are obtained for each extraction date.

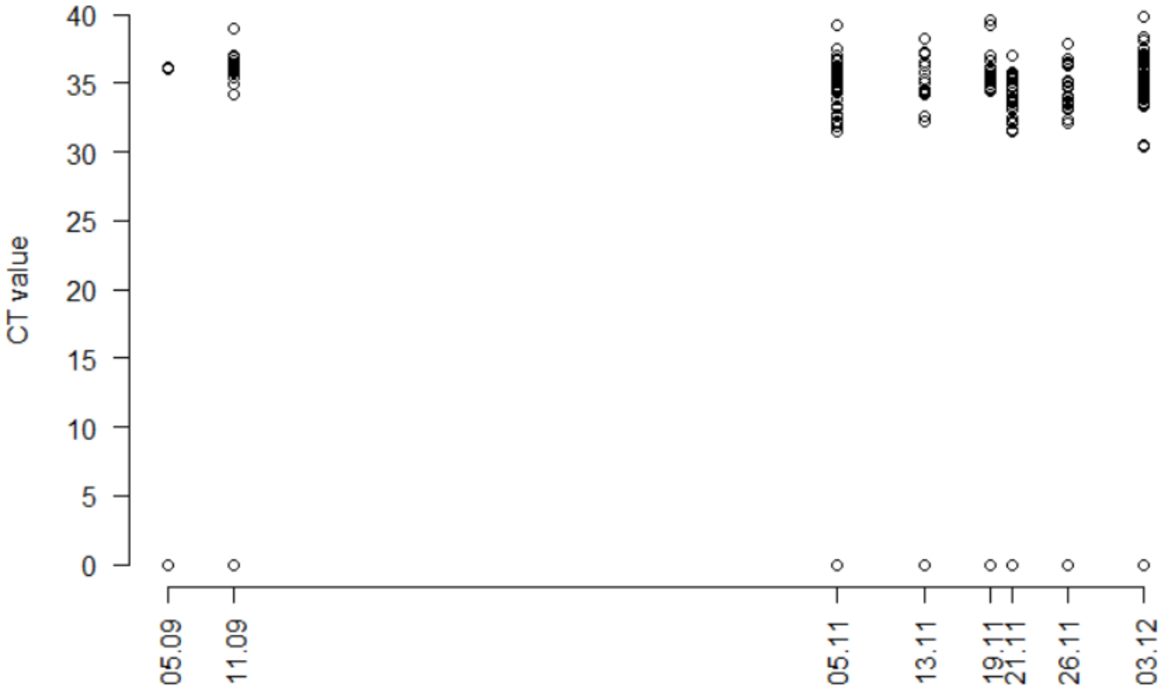


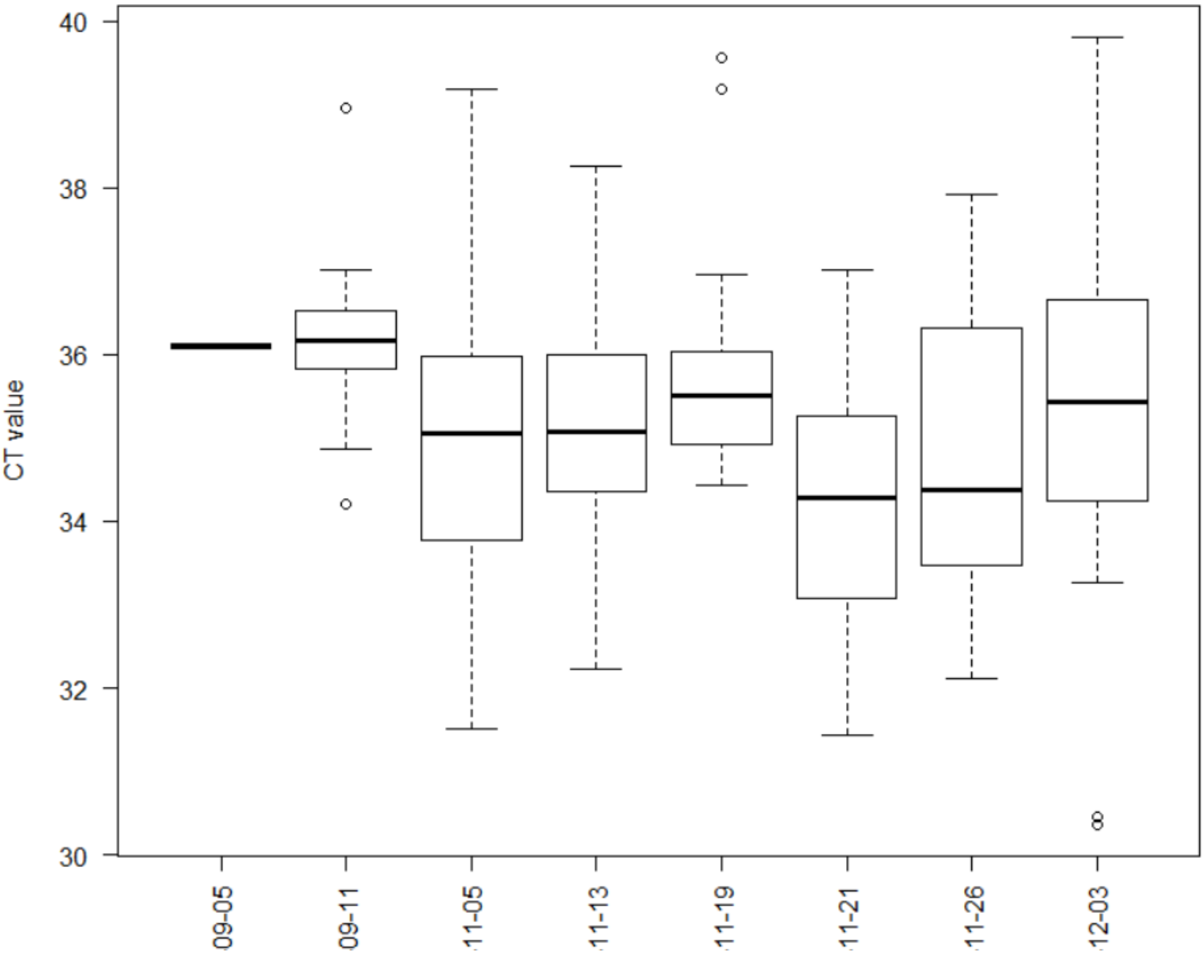

## References


Böhner J, Antonić O (2009). Chapter 8 Land-Surface Parameters Specific to Topo-Climatology. In: Hengl T, Reuter HI (eds) *Developments in Soil Science*, Geomorphometry. Elsevier Vol 33, pp 195–226.

Huete A, Justice C, Van Leeuwen W (1999). MODIS vegetation index (MOD13). Algorithm theoretical basis document. **3**.

Jarvis A (2008). Hole-field seamless SRTM data, International Centre for Tropical Agriculture (CIAT). *http://srtm csi cgiar org*.

Kopecký M, Čížková Š (2010). Using topographic wetness index in vegetation ecology: Does the algorithm matter? *Applied Vegetation Science* **13**: 450–459.

Murray FW (1966). *ON THE COMPUTATION OF SATURATION VAPOR PRESSURE.* RAND CORP SANTA MONICA CALIF.

OFS (2013). Waldmischungsgrad, Auflösung 100m: Geodaten | Publication. *Federal Statistical Office*.

OFS (2017). Statistique de la superficie selon nomenclature 2004 - Occupation du sol, description: métainformations sur les géodonnées | Publication. *Office fédéral de la statistique*.

O'Sullivan L, Bovet S, Streilein A (2008). TLM—the swiss 3D topographic landscape model. *ISPRS Proceeding* **37**: 1715–1719.

R Development Core Team (2008). R: A language and environment for statistical computing. *R Foundation for Statistical Computing*.

Riley SJ, DeGloria SD, Elliot R (1999). Index that quantifies topographic heterogeneity. *intermountain Journal of sciences* **5**: 23–27.

Running SW, Nemani RR, Hungerford RD (1987). Extrapolation of synoptic meteorological data in mountainous terrain and its use for simulating forest evapotranspiration and photosynthesis. *Canadian Journal of Forest Research* **17**: 472–483.

Thorne CR, Zevenbergen LW, Burt TP, Butcher DP (1987). Terrain analysis for quantitative description of zero-order basins. *IAHS-AISH publication*: 121–130.

Thornton PE, Running SW, White MA (1997). Generating surfaces of daily meteorological variables over large regions of complex terrain. *Journal of hydrology* **190**: 214–251.

Yokoyama R, Shirasawa M, Pike RJ (2002). Visualizing topography by openness: a new application of image processing to digital elevation models. *Photogrammetric engineering and remote sensing* **68**: 257–266.

Zimmermann NE, Roberts DW (2001). Final report of the MLP climate and biophysical mapping project. *Birmensdorf: Swiss Federal Research Institute*.
